# A wavelet lifting approach to long-memory estimation

**DOI:** 10.1007/s11222-016-9698-2

**Published:** 2016-09-03

**Authors:** Marina I. Knight, Guy P. Nason, Matthew A. Nunes

**Affiliations:** 1 Department of Mathematics, University of York, Heslington, York, YO10 5DD UK; 2 School of Mathematics, University of Bristol, Bristol, BS8 1TW UK; 3 Department of Mathematics and Statistics, Fylde College, Lancaster University, Lancaster, LA1 4YF UK

**Keywords:** Hurst exponent, Irregular sampling, Long-range dependence, Wavelets

## Abstract

Reliable estimation of long-range dependence parameters is vital in time series. For example, in environmental and climate science such estimation is often key to understanding climate dynamics, variability and often prediction. The challenge of data collection in such disciplines means that, in practice, the sampling pattern is either irregular or blighted by missing observations. Unfortunately, virtually all existing Hurst parameter estimation methods assume regularly sampled time series and require modification to cope with irregularity or missing data. However, such interventions come at the price of inducing higher estimator bias and variation, often worryingly ignored. This article proposes a new Hurst exponent estimation method which naturally copes with data sampling irregularity. The new method is based on a multiscale lifting transform exploiting its ability to produce wavelet-like coefficients on irregular data and, simultaneously, to effect a necessary powerful decorrelation of those coefficients. Simulations show that our method is accurate and effective, performing well against competitors even in regular data settings. Armed with this evidence our method sheds new light on long-memory intensity results in environmental and climate science applications, sometimes suggesting that different scientific conclusions may need to be drawn.

**Electronic supplementary material:**

The online version of this article (doi:10.1007/s11222-016-9698-2) contains supplementary material, which is available to authorized users.

## Introduction

Time series that arise in many fields, such as climatology (e.g. ice core data, Fraedrich and Blender 2003, atmospheric pollution, Toumi et al. 2001); finance, e.g. Jensen (1999) and references therein; geophysical science, such as sea level data analysis, Ventosa-Santaulària et al. (2014) and network traffic (Willinger et al., 1997), to name just a few, often display persistent (slow power-law decaying) autocorrelations even over large lags. This phenomenon is known as long memory or long-range dependence. Remarkably, the degree of persistence can be quantified by means of a single parameter, known in the literature as the Hurst parameter (Hurst 1951; Mandelbrot and Ness 1968). Estimation of the Hurst parameter leads, in turn, to the accurate assessment of the extent to which such phenomena persist over long time scales. This offers valuable insight into a multitude of modelling and analysis tasks, such as model calibration, trend detection and prediction (Beran et al. 2013; Vyushin et al. 2007; Rehman and Siddiqi 2009).

Data in many areas, such as climate science, are often difficult to acquire and hence will frequently suffer from omissions or be irregularly sampled. On the other hand, even data that is customarily recorded at regular intervals (such as in finance or network monitoring) often exhibit missing values which are due to a variety of reasons, such as equipment malfunction.

We first describe two examples that are shown to benefit from long-memory parameter estimation for irregularly spaced time series or series subject to missing observations, although our methods are, of course, more widely applicable.

### Long-memory phenomena in environmental and climate science time series

In climatology, the Hurst parameter facilitates the understanding of historical and geographical climate patterns or atmospheric pollution dynamics (Pelletier and Turcotte 1997; Fraedrich and Blender 2003), and consequent long-term health implications, for example.

In the context of climate modelling and simulation, Varotsos and Kirk-Davidoff (2006) write

*Models that hope to predict global temperature or total ozone over long time scales should be able to duplicate the long-range correlations of temperature and total ozone ...Successful simulation* [of long range correlations] *would enhance confidence in model predictions of climate and ozone levels.*

In particular, more accurate Hurst parameter estimation can also result in a better understanding of the origins of unexplained dependence behaviour from climate models (Tsonis et al. 1999; Fraedrich and Blender 2003; Vyushin et al. 2007).

**Fig. 1:**
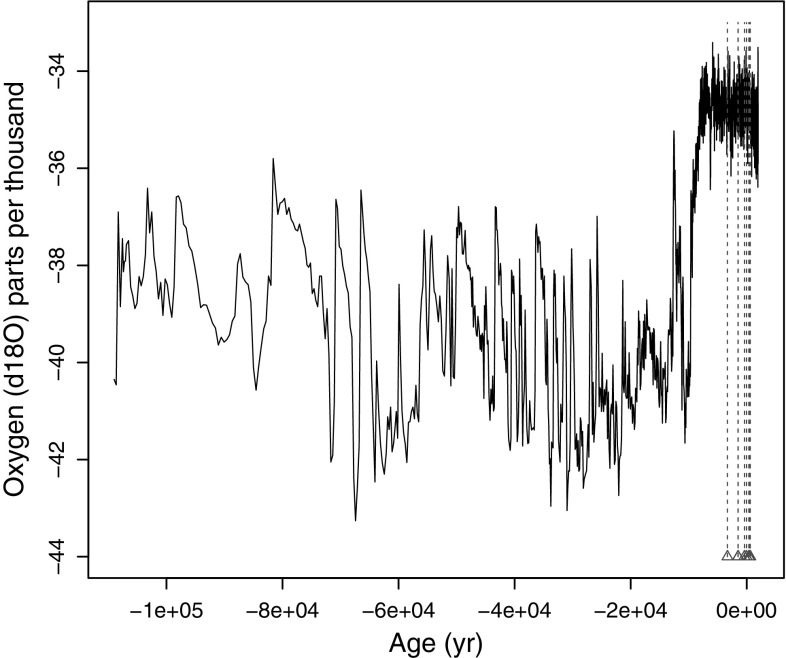
The $\delta ^{18}$O isotope record from the GISP2 ice core. *Triangles* indicate missing data locations, about 1 % near to the end of the series

*Isotopic cores* Ice core series are characterized by uneven time sampling due to variable geological pressure causing depletion and warping of ice strata, see e.g. Witt and Schumann (2005), Wolff (2005) or Vyushin et al. (2007) for a discussion of long-range dependence in climate science. We study an isotopic core series, where stable isotope levels measured through the extent of a core, such as $\delta ^{18}$O, are used as proxies representing different climatic mechanisms, for example, the hydrological cycle (Petit et al. 1999). Such data can indicate atmospheric changes occurring over the duration represented by the core (Meese et al. 1994). Here, long memory is indicative of internal ocean dynamics, such as warming/cooling episodes (Fraedrich and Blender 2003; Thomas et al. 2009). Such measures are used in climate models to understand present day climate variable predictability, including their possible response to global climate change (Blender et al. 2006; Rogozhina et al. 2011). Figure 1 shows $n=1403$ irregularly spaced oxygen isotopic ratios from the Greenland Ice Sheet Project 2 (GISP2) core; the series also features missing observations, indicated on the plot. For more details on these data, the reader is directed to e.g., Grootes et al. (1993); the data were obtained from the World Data Center for Paleoclimatology in Boulder, USA (http://www.ncdc.noaa.gov/paleo/icecore/).

*Atmospheric Pollutants* Long-range dependence quantification for air pollutants is widely considered in the literature, due to its relationship to the global atmospheric circulation and consequent climate system response, see e.g. Toumi et al. (2001), Varotsos and Kirk-Davidoff (2006), Kiss et al. (2007). Long-range dependence is also investigated for atmospheric measurements in e.g. Tsonis et al. (1999) and Tomsett and Toumi (2001). For atmospheric series in particular, such as ozone, underestimation of the long-range behaviour results in an underestimation of the frequency of weather anomalies, such as droughts (Pelletier and Turcotte 1997; Tsonis et al. 1999).

**Fig. 2:**
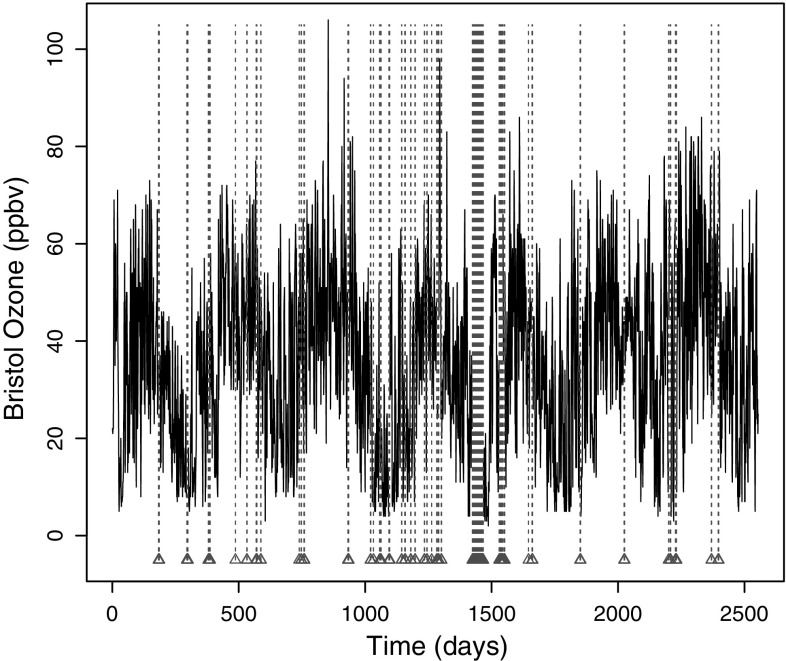
Ozone concentration (*ppbv*) at the Bristol Centre monitoring site. Missing locations indicated by *triangles*

Our data consist of average daily ozone concentrations measured over several years at six monitoring stations at Bristol Centre, Edinburgh Centre, Leeds Centre, London Bloomsbury, Lough Navar and Rochester. These sites correspond to an analysis of similar series in Windsor and Toumi (2001). Figure 2 shows the Bristol Centre series along with the locations of the missing concentration values. The percentage of missingness for the ozone series was in the range of 4–6 %. The data were acquired from the UK Department for Environment, Food and Rural Affairs UK-AIR Data Archive (http://uk-air.defra.gov.uk/).

### Aim and structure of the paper

A feature of many ice core series, such as that in Fig. 1, is that their sampling structure is naturally *irregular*. On the other hand, atmospheric series, such as the Ozone data in Fig. 2, are often designed to be measured at regular intervals, but can exhibit frequent dropout due to recording failures. In practice, a common way of dealing with these complex sampling structures is to aggregate (by temporal averaging) the series prior to analysis so that the data become regularly spaced (Clegg 2006). However, this has been shown to create spurious correlation and thus methods will tend to overestimate the memory persistence (Beran et al. 2013). Further evidence for inaccuracies in traditional estimation methods due to irregular or missing observations is given in Sect. 5.3. Similar overestimation has been observed when imputation or interpolation is used to mitigate for irregular or missing observations, see e.g. Zhang et al. (2014). In the context of climatic time series, this will consequently lead to misrepresenting feedback mechanisms in models of global climate behaviour, hence induce significant inaccuracy in forecasting weather variables or e.g. ozone depletion. Sections 6 and 7 discuss this in more detail.

Motivated by the lack of suitable long-memory estimation methods that deal naturally with sampling irregularity or missingness, which often occur in climate science data collection *and* by the grave scientific consequences induced by misestimation, we propose a novel method for Hurst parameter estimation suitable for time series with regular *or* irregular observations. Although the problems that spurred this work pertained to the environmental and climate science fields, our new method is general and flexible, and may be used for long-memory estimation in a variety of fields where the sampling data structure is complex, such as network traffic modelling (Willinger et al. 1997).

Wavelet-based approaches have proved to be very successful in the context of regularly sampled long-memory time series (for details see Sect. 2) and are the ‘right domain’, Flandrin (1998), in which to analyze them. For irregularly sampled processes, or those featuring missingness, we propose the use of the lifting paradigm (Sweldens 1995) as the version of the classical wavelet transform for such data. In particular, we select the nondecimated lifting transform proposed by Knight and Nason (2009) which has been recently shown to perform well for other time series tasks, such as spectral analysis, in Knight et al. (2012). Whilst dealing naturally with the irregularity in the time domain, our method is shown to also yield competitive results for regularly spaced data, thus extending its applicability.

Section 2, next, reviews long-memory processes and provides an overview of lifting and the nondecimated wavelet lifting transform. Section 3 explains how lifting decorrelates long-memory series and Sect. 4 shows how this can be exploited to provide our new lifting-based Hurst exponent estimation procedure. Section 5 provides a comprehensive performance assessment of our new method via simulation. Section 6 demonstrates our technique on the previously introduced data sets and discusses the implication of its results for each set. Section 7 concludes this work with discussion and some ideas for future exploration.

## Review of long-range dependence, its estimation, wavelets and lifting

Long-range behaviour is often characterized by a parameter, such as the Hurst exponent, *H*, introduced to the literature by Hurst (1951) in hydrology. Similar concepts were discussed by the pioneering work of Mandelbrot and Ness (1968) that introduced self-similar and related processes with long memory, including statistical inference for long-range dependent processes. A large body of statistical literature has since grown dedicated to the estimation of *H*. Reviews of long memory can be found in Palma (2007) or Beran et al. (2013).

Time domain *H* estimation methods include the R/S statistic (Mandelbrot and Taqqu 1979; Bhattacharya et al. 1983); aggregate series variance estimators (Taqqu et al. 1995; Teverovsky and Taqqu 1997; Giraitis et al. 1999); least squares regression using subsampling in Higuchi (1990); variance of residuals estimators in Peng et al. (1994).

Frequency domain estimators of *H* include Whittle estimators, see Fox and Taqqu (1986), Dahlhaus (1989), and connections to Fourier spectrum decay are made in e.g. Lobato and Robinson (1996). Long-memory time series have wavelet periodograms exhibiting similar log-linear relationships to the Hurst exponent, see for example McCoy and Walden (1996). Wavelet-based regression approaches such as Percival and Guttorp (1994), Abry et al. (1995), Abry et al. (2000) and Jensen (1999) have been shown to be successful. Stoev et al. (2004) and Faÿ et al. (2009) provide complete investigations of frequency-based estimators. Extensions of wavelet estimators to other settings, for example the presence of observational noise, can be found in Stoev et al. (2006), Gloter and Hoffmann (2007). Other recent works concerning long-memory estimation including multiscale approaches are Vidakovic et al. (2000), Shi et al. (2005), Hsu (2006), Jung et al. (2010), Coeurjolly et al. (2014) and Jeon et al. (2014). Reviews comparing several techniques for Hurst exponent estimation can be found in e.g. Taqqu et al. (1995).

A shortcoming of the approaches above is that they are inappropriate, and usually not robust, in the irregularly spaced/missing observation situation. Treating such data with the usual practical ‘preprocessing’ approach of imputation, interpolation and/or aggregation induces high estimator bias and errors, as highlighted by Clegg (2006), Beran et al. (2013) and Zhang et al. (2014), for example. The implicit danger is that such preprocessing may inadvertently change the conclusions of subsequent scientific modelling and prediction, e.g. see Varotsos and Kirk-Davidoff (2006).

A possible solution might be to estimate the Hurst parameter directly from a spectrum estimated on irregular data. For example, the Lomb-Scargle periodogram, (Lomb 1976; Scargle 1982), estimates the spectrum from irregularly spaced data. In the context of stationary processes, the Lomb-Scargle periodogram has been shown to correctly identify peaks but to overestimate the spectrum at high frequencies (Broersen 2007), while Rehfeld et al. (2011) and Nilsen et al. (2016) argue that irregularly sampled data cause various problems for all spectral techniques. In particular, they report that severe bias arises in the Lomb-Scargle periodogram if there are no periodic components underlying the true spectra [e.g. turbulence data, Broersen et al. (2000)]. The weighted wavelet *Z*-transform construction of Foster (1996) also reinforces this point, and is subsequently successfully used for describing fractal scaling behaviour by Kirchner and Neal (2013). A theoretical and detailed empirical study of Hurst estimation via this route would be an interesting avenue for further study, but not pursued further here.

### Long-range dependence (LRD)

Long-memory processes $X=\{ X(t), t \in {\mathbb {R}}\}$ are stationary finite variance processes whose spectral density satisfies $f_{X}(\omega ) \sim c_f |\omega |^{-\alpha }$ for frequencies $\omega \rightarrow 0$ and $\alpha \in (0,1)$, or, equivalently, whose autocovariance $\gamma _X(\tau ) \sim c_{\gamma } \tau ^{-\beta }$ as $\tau \rightarrow \infty$ and $\beta =1-\alpha \in (0,1)$, where $\sim$ means asymptotic equality. The parameter $\alpha$ controls the intensity of the long-range behaviour.

The Hurst exponent, *H*, naturally arises in the context of self-similar processes with self-similarity parameter *H*, which satisfy $X (at) \overset{d}{=} a^H X(t)$ for $a>0$, $H \in (0,1)$ and where $\overset{d}{=}$ means equal in distribution. Self-similar processes, while obviously non-stationary, can have stationary increments and the variance of such processes is proportional to $|t|^{2H}$, with $H \in (0,1)$. The stationary increment process of a self-similar process with parameter *H* has been shown to have long memory when $0.5<H<1$, and the two parameters $\alpha$ and *H* are related through $\alpha =2H-1$. In general, if $0.5<H<1$ the process exhibits long memory, with higher *H* values indicating longer memory, whilst if $0<H<0.5$ the process has short memory. The case of $H=0.5$ represents white noise.

Examples of such processes are fractional Brownian motion, its (stationary) increment process, fractional Gaussian noise, and fractionally integrated processes. Fractionally integrated processes *I*(*d*), (Granger and Joyeux 1980), are characterized by a parameter $d\in (-1/2,1/2)$ which dictates the order of decay in the process covariance and has long memory when $d>0$, with the relationship to the Hurst exponent *H* given by $H=d+1/2$. Abry et al. (2000) and Jensen (1999) showed that *H*, *d* and the spectral power decay parameter, $\alpha$ are linearly related.

### Existing wavelet-based estimation of long memory

Much contemporary research on long-memory parameter estimation relies on wavelet methods and produce robust, reliable, computationally fast and practical estimators—see, for example, McCoy and Walden (1996), Whitcher and Jensen (2000) and Ramírez-Cobo et al. (2011). Long-memory wavelet estimators (of *H*, *d* or $\alpha$) base estimation on the *wavelet spectrum*, the wavelet equivalent of the Fourier spectral density, see Vidakovic (1999) or Abry et al. (2013) for more details.

Specifically, suppose a *discrete* series $\{X_t\}_{t=0}^{N-1}$ has long-memory parameter $\alpha$. Assuming regular time sampling, a wavelet estimate of $\alpha$ can be obtained by:

1. Perform the discrete wavelet transform (DWT) of $\{X_t\}_{t=0}^{N-1}$ to obtain wavelet coefficients, $\{ d_{j, k} \}_{j, k}$, where $j = 1, \ldots , J$ is the coefficient scale and $k = 1, \ldots , n_j=2^j$ its time location. It can be shown that, e.g. Stoev et al. (2004), the wavelet energy
  1 $$\begin{aligned} {\mathbb {E}}(d_{j,k}^2) \sim \hbox {const} \times 2^{j\alpha },\ \forall \ k \quad \hbox {as } \ j \longrightarrow \infty . \end{aligned}$$
2. Estimate the wavelet energy within each scale *j* by $e_j = n^{-1}_j\sum _{k=1}^{n_j}d_{j,k}^2$.
3. The slope of the linear regression fitted to a subset of $\{( j,\hbox {log}_{2} e_j) \}_{j=1}^J$ estimates $\alpha$, see Beran et al. (2013) for details.

Later, we show that methods designed for regularly spaced data often fail to deliver a robust estimate if the time series is subject to missing observations or has been sampled irregularly. Much literature is silent on the issue of how to estimate Hurst when faced with irregular or missing data. One possible, and often quoted, solution is to aggregate data into regularly spaced bins, but no warnings are usually provided for its pitfalls, see Sect. 5.3 for further information. Our solution to this problem is to build an estimator out of coefficients obtained from a (lifting) wavelet transform designed for irregularly sampled observations, as described next.

### Wavelet lifting transforms for irregular data

The *lifting algorithm* was introduced by Sweldens (1995) to provide ‘second-generation’ wavelets adapted for intervals, domains, surfaces, weights and irregular samples. Lifting has been used successfully for nonparametric regression problems and spectral estimation with irregularly sampled observations, see e.g., Trappe and Liu (2000), Nunes et al. (2006), Knight and Nason (2009) and Knight et al. (2012). Jansen and Oonincx (2005) give a recent review of lifting.

Our Hurst exponent estimation method makes use of a recently developed lifting transform called the *lifting one coefficient at a time* (LOCAAT) transform proposed by Jansen et al. (2001, (2009) which works as follows.

Suppose a function $f(\cdot )$ is observed at a set of *n*, possibly irregular, locations or time points, $\underline{x}=(x_{1},\, \ldots , \, x_{n})$ and represented by $\{(x_{i},f(x_i)=f_{i})\}_{i=1}^{n}$. LOCAAT starts with the $\underline{f} = (f_{1},\, \ldots , \, f_{n})$ values which, in wavelet nomenclature, are the initial so-called scaling function values. Further, each location, $x_i$, is associated with an interval which it intuitively ‘spans’. For our problem, the interval associated with $x_i$ encompasses all continuous time locations that are closer to $x_i$ than any other location—the Dirichlet cell. Areas of densely sampled time locations are thus associated with sets of shorter intervals. The LOCAAT algorithm, as designed in Jansen et al. (2009), has both the initial and dual scaling basis functions given by suitably scaled characteristic functions over these intervals, but, in general, this is not a requirement.

The aim of LOCAAT is to transform the initial $\underline{f}$ into a set of, say, *L* coarser scaling coefficients and $(n-L)$ wavelet-like coefficients, where *L* is a desired ‘primary resolution’ scale.

Lifting works by repeating three steps: split, predict and update. In LOCAAT, the *split* step consists in choosing a point to be lifted. Once a point, $j_n$, has been selected for removal, denoted $(x_{j_{n}},f_{j_{n}})$, we identify its set of neighbouring observations, ${\mathscr {I}}_{n}$. The *predict* step estimates $f_{j_{n}}$ by using regression over the neighbouring locations ${\mathscr {I}}_{n}$. The prediction error (the difference between the true and predicted function values), $d_{j_{n}}$ or detail coefficient, is then computed by

2 $$\begin{aligned} d_{j_{n}}=f_{j_{n}}-\sum _{i\in {\mathscr {I}}_{n}}a^{n}_{i}f_{i}, \end{aligned}$$

where $(a^{n}_{i})_{i\in {\mathscr {I}}_{n}}$ are the weights resulting from the regression procedure over ${\mathscr {I}}_{n}$. For example, in the simplest single neighbour case this reduces to $d_{j_{n}}=f_{j_{n}}-f_{i}$.

In the *update* step, the *f*-values of the neighbours of $j_n$ are updated by using a weighted proportion of the detail coefficient:

3 $$\begin{aligned} f_{i}^{({ updated})}:=f_{i}+b^{n}_{i}d_{j_{n}},\quad i\in {{\mathscr {I}}_{n}}, \end{aligned}$$

where the weights $(b^{n}_{i})_{i\in {\mathscr {I}}_{n}}$ are obtained from the requirement that the algorithm preserves the signal mean value (Jansen et al. 2001, 2009). The interval lengths associated with the neighbouring points are also updated to account for the decreasing number of unlifted coefficients that remain. This redistributes the interval associated to the removed point to its neighbours. The three steps are then repeated on the updated signal, and after each repetition a new wavelet coefficient is produced. Hence, after say $(n-L)$ removals, the original data is transformed into *L* scaling and $(n-L)$ wavelet coefficients. LOCAAT is similar in spirit to the classical DWT step which takes a signal vector of length $2^\ell$ and through separate local averaging and differencing-like operations produces $2^{\ell -1}$ scaling and $2^{\ell -1}$ wavelet coefficients.

As LOCAAT progresses, scaling and wavelet functions decomposing the frequency content of the signal are built recursively according to the predict and update Eqs. (2) and (3). Also, the (dual) scaling functions are defined recursively as linear combinations of (dual) scaling functions at the previous stage. To aid description of our Hurst exponent estimation method in Sects. 3 and 4, we recall the recursion formulas for the (dual) scaling and wavelet functions at lifting stage *r*:

4 $$\begin{aligned}&\tilde{\varphi }_{r-1,i}(x)=\tilde{\varphi }_{r,i}(x)+b_i^r \tilde{\psi }_{j_r}(x), \quad i\in {\mathscr {I}}_r \end{aligned}$$

5 $$\begin{aligned}&\tilde{\varphi }_{r-1,i}(x)=\tilde{\varphi }_{r,i}(x), \quad i \notin {\mathscr {I}}_r \end{aligned}$$

6 $$\begin{aligned}&\tilde{\psi }_{j_r}(x)=\tilde{\varphi }_{r,j_r}(x)-\sum _{i \in {\mathscr {I}}_r}a_i^r \tilde{\varphi }_{r,i}(x). \end{aligned}$$

After $(n-L)$ lifting steps, the signal $\underline{f}$ can be expressed as the linear combination

7 $$\begin{aligned} f(x)=\sum _{r=L+1}^{n} d_{j_{r}}\psi _{j_{r}}(x)+ \mathop { \sum _{i\in \{1, \ldots , n\}\setminus }}_{ \{j_{n},j_{n-1},\ldots ,j_{L+1}\}} c_{L,i}\varphi _{L,i}(x), \end{aligned}$$

where $\psi _{j_{r}}(x)$ is a wavelet function representing high frequency components and $\varphi _{L,i}(x)$ is a scaling function representing the low frequency content. Just as in the classical wavelet case, the detail coefficients can be synthesized by means of the (dual) wavelet basis, e.g. $d_{j_r}=\langle f, \tilde{\psi }_{j_r}\rangle$, where $\langle \cdot , \cdot \rangle$ denotes the $L^2$-inner product.

A feature of lifting, hence also of LOCAAT, is that the forward transform can be inverted easily by reversing the split, predict and update steps.

*Artificial wavelet levels* The notion of scale for second generation wavelets is continuous, which indirectly stems from the fact that second generation wavelets are not dyadically scaled versions of a single mother wavelet. To mimic the dyadic levels of classical wavelets, Jansen et al. (2009) group wavelet functions of similar (continuous) scales into ‘artificial’ levels. Similar results are also obtained by grouping the coefficients via their interval lengths into ranges $(2^{j-1}\alpha _0,2^{j}\alpha _0]$, where $j \ge 1$ and $\alpha _0$ is the minimum scale. This construction is more evocative of the classical wavelet dyadic scales.

*Choice of removal order* In the DWT the finest scale coefficients are produced first and followed by progressively coarser scales. Jansen et al. (2009) mimic this behaviour by removing points in order from the finest continuous scale to the coarsest. However, the LOCAAT scheme can accommodate any coefficient removal order. In particular, we can choose to remove points following a predefined *path* (or *trajectory*) $T=(x_{o_{1}}, \, \ldots ,\,x_{o_{n}})$, where $(o_{1}, o_{2},\, \ldots ,\, o_{n})$ is a permutation of the set $\{1, \, \ldots , \,n\}$. Knight and Nason (2009) introduced the nondecimated lifting transform which explores the space of *n*! possible trajectories via bootstrapping. The nondecimated lifting transform resembles the nondecimated wavelet transform (Coifman and Donoho 1995; Nason and Silverman 1995) in that both are designed to mitigate the effect of poor performance caused by the relative location of signal features and wavelet position. Our technique in Sect. 4 below also exploits the trajectory space via bootstrapping, in order to improve the accuracy of our Hurst exponent estimator.

## Decorrelation properties of the LOCAAT algorithm

Wavelet transforms are known to possess good compression and decorrelation properties. For long-memory processes this has been shown for the discrete wavelet transform by, e.g., Vergassola and Frisch (1991) and Flandrin (1992) for fractional Brownian motion, Abry et al. (2000) for fractional Gaussian noise, Jensen (1999) for fractionally integrated processes, Craigmile et al. (2001) for fractionally differenced processes or, for a more general discussion, see e.g. Vidakovic (1999, Chap. 9) or Craigmile and Percival (2005). Whilst lifting has repeatedly shown good performance in nonparametric regression and spectral estimation problems, a rigorous theoretical treatment is often difficult due to the irregularity and lack of the Fourier transform in this situation.Some lifting transforms have been shown to have good decorrelation properties, see Trappe and Liu (2000) or Claypoole et al. (1998) for further details on their compression abilities.

Decorrelation is important for long-memory parameter estimation as taking the wavelet transform produces coefficients that are “quasidecorrelated,” see Flandrin (1992) and Veitch and Abry (1999), Property P2, page 880. The decorrelation, and consequent removal of the long memory, then permits the use of established methods for long-memory parameter estimation using the lifting coefficients. Next, we provide analogous mathematical evidence for the LOCAAT decorrelation properties which benefit our Hurst parameter estimation procedure presented later in Sect. 4. It is important to realize that although the statement of Proposition 1 is visually similar to earlier ones concerning regular wavelets, such as Abry et al. (2000, p.51) for fractional Gaussian noise, Jensen (1999, Theorem 2) for fractionally integrated processes or Theorem 5.1 of Craigmile and Percival (2005) for fractionally differenced processes, our proposition establishes the result for the lifting transform, which is considerably more challenging than for regular wavelets involving new mathematics.

**Fig. 3:**
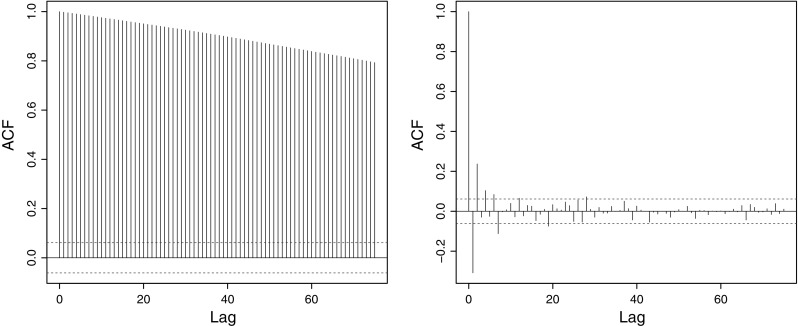
Decorrelation properties of LOCAAT. *Left* simulated fractional Brownian motion autocorrelation with $H=0.9$. *Right* the autocorrelation after LOCAAT transformation

### Theoretical decorrelation due to lifting for stationary long-memory series

#### Proposition 1

Let $X = \{X_{t_i}\}_{i=0}^{N-1}$ denote a (zero-mean) stationary long-memory time series with Lipschitz continuous spectral density $f_{X}$. Assume the process is observed at irregularly spaced times $\{t_i\}_{i=0}^{N-1}$ and let $\{ \{ c_{L,i}\}_{i\in \{0, \ldots , N-1\} \setminus \{j_{N-1},\ldots ,j_{L-1}\} } , \{ d_{j_r} \}_{r=L-1}^{N-1} \}$ be the LOCAAT transform of *X*. Then the detail coefficients $\{ d_{j_r} \}_{r}$ have autocorrelation with rate of decay faster than any process with long memory with autocorrelation decay $\tau ^{-\beta }$ for $\beta \in (0,1)$.

The proof can be found in Appendix A. Proposition 1 assumes no specific lifting wavelet. We conjecture that if smoother lifting wavelets were employed, it might be possible to obtain even better rates of decay for the lifting coefficients’ autocorrelations along similar lines to the equivalent result for classical wavelets shown by Abry et al. (2000). To complement our mathematical result we next investigate decorrelation of a nonstationary self-similar process with long-memory increments via simulation.

### Empirical decorrelation due to lifting for nonstationary self-similar processes

We simulated $K=100$ regularly sampled fractional Brownian motion (FBM) series $\{X_t\}^{(l)}$ ($l=1,\ldots ,K$) of length $n=2^j$ for six *j* ranging from 8 to 13 with true Hurst parameters *H* ranging from 0.6 to 0.9. The series were generated using the *fArma* R add-on package (Wuertz et al. 2013).

Figure 3 illustrates the powerful decorrelation effect of LOCAAT when applied to a single fractional Brownian motion realization of length $n=1024$ with Hurst parameter $H=0.9$. The left-hand plot clearly shows the characteristic slow decay of long memory whereas the right-hand plot shows only small short term correlation after LOCAAT application in the first six or seven lags. To assess the overall decorrelation ability we compute the mean relative absolute autocorrelation

8 $$\begin{aligned} {\hbox {REL}_{ac}} = 100 K^{-1} \sum _{l=1}^{K} \frac{\sum _{ r \ne k}|{\text {Cov}}(d^{(l)}_{j_r},d^{(l)}_{j_k})|}{\sum _{ i \ne j}|{\text {Cov}}(X^{(l)}_{t_i},X^{(l)}_{t_j})|}, \end{aligned}$$

where $\underline{d}^{(l)}$ is the LOCAAT-transformed $\{X_t\}^{(l)}$; hence a small percentage ${\hbox {REL}_{ac}}$ value means that LOCAAT performed highly effective decorrelation. Table 1 shows the efficacious decorrelation results for the various fractional Brownian processes. The mean relative absolute autocorrelation has been reduced by at least 95 % on the average for all situations and by 99 % for $n \ge 2048$.

**Table 1:** Mean relative absolute autocorrelation (%) for simulated fractional Brownian motion

H	Series length, *n*					
	256	512	1024	2048	4096	8192
0.6	4.5	2.3	1.4	0.8	0.5	0.2
0.7	3.6	2.1	1.2	0.5	0.3	0.2
0.8	3.0	1.5	0.9	0.4	0.2	0.1
0.9	2.4	1.3	0.7	0.3	0.2	0.1

## Long-memory parameter estimation using wavelet lifting (LoMPE)

We now show that the $\log _2$-variance of the lifting coefficients is linearly related to the artificial scale level which parallels the classical wavelet result in (1). This new result enables *direct* construction of a simple Hurst parameter estimator for irregularly sampled time series data. As with Proposition 1, the statement of Proposition 2 is visually similar to that for established results in the literature corresponding to regular wavelets. However, again, the proof of our proposition relies on new mathematics for the more difficult situation of lifting.

### Proposition 2

Let $X=\{X_{t_i}\}_{i=0}^{N-1}$ denote a (zero-mean) long-memory stationary time series with finite variance and spectral density $f_{X}(\omega ) \sim c_f |\omega |^{-\alpha }$ as $\omega \rightarrow 0$, for some $\alpha \in (0,1)$. Assume the series is observed at irregularly spaced times $\{t_i\}_{i=0}^{N-1}$ and transform the observed data *X* into a collection of lifting coefficients, $\{ d_{j_r} \}_r$, via application of LOCAAT from Sect. 2.3.

Let *r* denote the stage of LOCAAT at which we obtain the wavelet coefficient $d_{j_r}$, and let its corresponding artificial level be $j^\star$, then for some constant *K*

9 $$\begin{aligned} \sigma ^2_{j^{\star }} = {\text {Var}}({d}_{j_r}) \sim 2^{j^{\star }(\alpha - 1)} \times K. \end{aligned}$$

The proof can be found in Appendix A. We now use this result to suggest a long-memory parameter estimation method from an irregularly sampled time series.

**Fig. 4:**
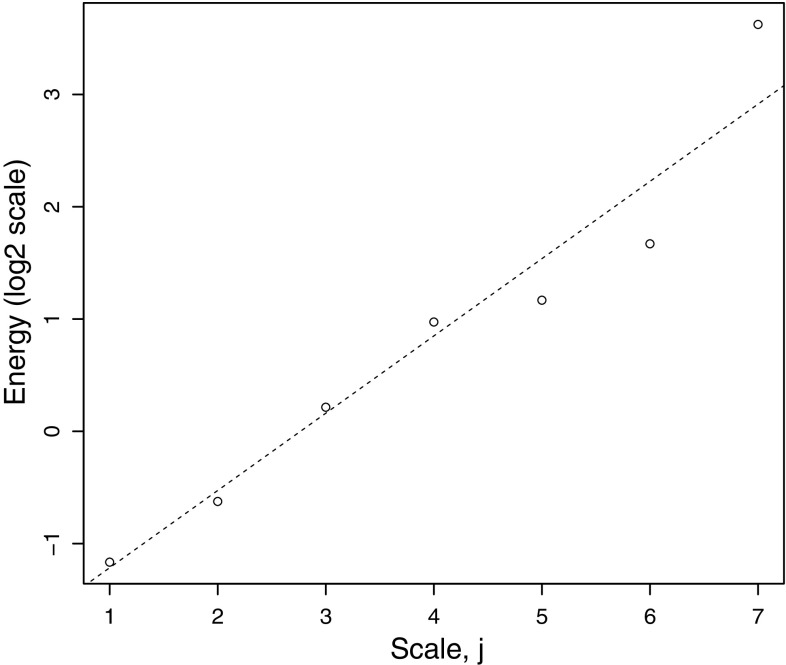
$\hbox {Log}_2$ of estimated wavelet coefficient variances $\hat{\sigma }^2_{j{}_{\phantom {0_0}}}$ versus scale, computed on fractional Gaussian noise series of length $N=1024$ with Hurst parameter of $\alpha = 0.8$ and 10 % missingness at random. Estimated Hurst parameter from weighted regression slope is $\hat{\alpha } = 0.84$

Long-Memory Parameter Estimation Algorithm (LoMPE)

Assume that $\{X_{t_i}\}_{i=0}^{N-1}$ is as in Proposition 2. We estimate $\alpha$ as follows.

1. Apply LOCAAT to the observed process $\{X_{t_i}\}_{i=0}^{N-1}$ using a particular lifting trajectory to obtain lifting coefficients $\{ d_{j_r} \}_r$. Then group the coefficients into a set of artificial scales as described in Sect. 2.3.
2. Normalize the detail coefficients by dividing through by the square root of the corresponding diagonal entry of $\tilde{W}\tilde{W}^T$, where $\tilde{W}$ is the lifting transform matrix. To avoid notational clutter we continue to use $d_{j_r}$ to denote the normalized details, $d_{j_r} (\tilde{W}\tilde{W}^T)^{-1/2}_{j_r, j_r}$.
3. Estimate the wavelet coefficients’ variance within each artificial level $j^{\star }$ by
  10 $$\begin{aligned} \qquad \hat{\sigma }^2_{j^{\star }} := ( n_{j^\star }-1)^{-1} \sum _{r =1}^{n_{j^\star }}d_{j_r}^2, \end{aligned}$$
  where $n_{j^{\star }}$ is the number of observations in artificial level $j^{\star }$.
4. Fit a weighted linear regression to the points $\log _2 ( \hat{\sigma }^2_{j^{\star }} )$ versus ${j^{\star }}$; use its slope to estimate $\alpha$.
5. Repeat steps A-1 to A-4 for *P* bootstrapped trajectories, obtaining an estimate $\hat{\alpha }_p$ for each trajectory $p \in \overline{1,P}$. The final estimator is $\hat{\alpha }=P^{-1} \sum _{p=1}^P{\hat{\alpha }_p}.$

As an example, Fig. 4 plots the $\log _2$-wavelet variances versus artificial scale resulting from the above algorithm being applied to a simulated fractional Gaussian noise series. It is clear from the plot that the $\log _2$-variances are well modelled by a straight line even in this case where the noise series suffers from dropout of 10 % missing-at-random.

### Remark 1

The normalization in step A-2 corrects for the lack of orthonormality inherent in the lifting transform ($\tilde{W})$.

### Remark 2

We use the simple additive formula (10) in step A-3 as the detail coefficients have zero mean and small correlation due to the effective decorrelation properties of the LOCAAT transform observed in Sect. 3.

### Remark 3

As , we correct for the bias introduced by regressing  quantities in step A-4 using the same weighting as proposed by Veitch and Abry (1999), hence accounting for the different variability across artificial levels. The weights are obtained under the Gaussianity assumption, though Veitch and Abry (1999) report insensitivity to departures from this assumption.

### Remark 4

The approach in step A-5 is similar to model averaging over different possible wavelet bases (cycle-spinning) as proposed by Coifman and Donoho (1995) and adapted to the lifting context by Knight and Nason (2009). Averaging over the different wavelet bases improves the variance estimation and mitigates for ‘abnormal trajectories’. If an estimate $\hat{\alpha }$ is obtained by means of regression without variance weighting, our approach yields a reasonable confidence interval without relying on the Gaussianity assumption, as in Abry et al. (2000). Trajectories are randomly drawn, where each removal order is generated by sampling $(N-L)$ locations without replacement from $\{ t_i \}_{i=0}^{N-1}$.

## Simulated performance of LoMPE

Our simulation study is intended to reflect many real-world data scenarios. The simulated time series should be long enough to be able to reasonably estimate what is, after all, a low-frequency asymptotic quantity. For example, Clegg (2006) uses 100000 observations, which is maybe somewhat excessive, whereas Jensen (1999) examines the range $2^7$–$2^{10}$. We investigated processes of lengths of 256, 512 and 1024. Although our method does not require a dyadic number of observations, dyadic process lengths have been chosen to ensure comparability with classical wavelet methods in regular settings.

**Table 2:** Mean squared error ($\times 10^3$) for regularly spaced fractional Brownian motion series for a range of Hurst parameters for the estimation procedures described in the text

*H*	$n=256$			$n=512$			$n=1024$		
	Peng	Wavelet	LoMPE	Peng	Wavelet	LoMPE	Peng	Wavelet	LoMPE
0.6	19 (30)	29 (48)	$\boxed {12}$ (21)	13 (22)	20 (37)	$\boxed {9}$ (15)	$\boxed {10}$ (14)	13 (20)	$\boxed {10}$ (11)
0.7	25 (35)	34 (57)	$\boxed {12}$ (15)	14 (16)	21 (34)	$\boxed {8}$ (11)	9 (12)	15 (24)	$\boxed {8}$ (9)
0.8	19 (23)	24 (45)	$\boxed {11}$ (13)	13 (18)	17 (28)	$\boxed {7}$ (10)	12 (16)	15 (22)	$\boxed {8}$ (10)
0.9	$\boxed {23}$ (39)	34 (69)	28 (39)	15 (23)	17 (31)	$\boxed {13}$ (20)	12 (16)	16 (26)	$\boxed {7}$ (9)

Numbers in brackets represent the standard deviation of estimation errors. Boxed numbers indicate best result

**Table 3:** Mean squared error ($\times 10^3$) for regularly spaced fractional Gaussian noise for a range of Hurst parameters for the estimation procedures described in the text

*H*	$n=256$			$n=512$			$n=1024$		
	Peng	Wavelet	LoMPE	Peng	Wavelet	LoMPE	Peng	Wavelet	LoMPE
0.6	8 (11)	31 (50)	$\boxed {2}$ (2)	4 (6)	11 (19)	$\boxed {1}$ (1)	2 (3)	8 (13)	$\boxed {1}$ (1)
0.7	7 (8)	27 (49)	$\boxed {2}$ (3)	3 (5)	12 (19)	$\boxed {1}$ (1)	3 (3)	9 (15)	$\boxed {1}$ (1)
0.8	7 (11)	29 (70)	$\boxed {2}$ (3)	5 (6)	16 (26)	$\boxed {2}$ (3)	4 (6)	10 (16)	$\boxed {3}$ (2)
0.9	10 (13)	28 (64)	$\boxed {3}$ (4)	4 (5)	11 (15)	$\boxed {2}$ (3)	$\boxed {3}$ (5)	10 (17)	4 (2)

Numbers in brackets represent the standard deviation of estimation errors. Boxed numbers indicate best result

**Table 4:** Mean squared error ($\times 10^3$) for regularly spaced fractionally integrated series for a range of Hurst parameters, $H=d+1/2$, for the estimation procedures described in the text

*H*	$n=256$			$n=512$			$n=1024$		
	Peng	Wavelet	LoMPE	Peng	Wavelet	LoMPE	Peng	Wavelet	LoMPE
0.6	8 (9)	25 (39)	$\boxed {3}$ (4)	4 (6)	16 (39)	$\boxed {1}$ (2)	2 (2)	8 (13)	$\boxed {1}$ (1)
0.7	8 (11)	29 (39)	$\boxed {4}$ (5)	$\boxed {4}$ (5)	9 (15)	$\boxed {4}$ (4)	$\boxed {3}$ (3)	6 (10)	4 (3)
0.8	11 (16)	28 (39)	$\boxed {6}$ (8)	7 (8)	18 (34)	$\boxed {6}$ (5)	$\boxed {4}$ (5)	6 (11)	6 (4)
0.9	12 (15)	30 (53)	$\boxed {7}$ (8)	$\boxed {7}$ (10)	11 (18)	8 (7)	$\boxed {4}$ (6)	8 (14)	9 (5)

Numbers in brackets represent the standard deviation of estimation errors. Boxed numbers indicate best result

To investigate the effect of missing observations on the performance of our method, we simulated datasets with an increasing level of random missingness (5–20 %). This reflects real data scenarios, as documented by current literature that deals with time series analysis under the presence of missingness, e.g. paleoclimatic data (Broersen 2007), such as the isotopic cores, and air pollutant data (Junger and Ponce de Leon 2015).

We compared results across the usual range of Hurst parameters $H=0.6, \ldots , 0.9$ for fractional Brownian motion, fractional Gaussian noise and fractionally integrated series. The processes were simulated via the *fArma* add-on package (Wuertz et al. 2013) for the R statistical programming language (Core Team 2013). Each set of results is taken over $K=100$ realizations and $P=50$ lifting trajectories (denoted “*LoMPE*”), using modifications to the code from the *adlift* package (Nunes and Knight 2012) and the *nlt* package (Knight and Nunes 2012). The simulations were repeated for two competitor methods: the wavelet-based regression technique of McCoy and Walden (1996), Jensen (1999), optimized for the choice of wavelet (denoted “*wavelet*”), as well as the residual variance method (Peng et al. 1994), which we denote “*Peng*”. Both methods are available in the *fArma* package and were chosen as our empirical results indicated that these techniques performed the best amongst traditional methods over a range of simulation settings.

### Performance for regularly sampled series

For the simulations described above, Tables 2, 3 and 4 report the mean squared error (MSE) defined by

11 $$\begin{aligned} \hbox {MSE} = K^{-1} \sum _{k=1}^{K} (H-\hat{H}^{k})^2. \end{aligned}$$

**Table 5:** Mean squared error ($\times 10^3$) for irregularly spaced fractional Brownian motion series featuring different degrees of missing observations for a range of Hurst parameters for the LoMPE estimation procedure

*H*	$n=256$			$n=512$			$n=1024$		
	Missingness proportion, *p*			Missingness proportion, *p*			Missingness proportion, *p*		
	5 %	10 %	20 %	5 %	10 %	20 %	5 %	10 %	20 %
0.6	13 (22)	14 (23)	16 (25)	11 (16)	12 (17)	13 (19)	12 (12)	13 (13)	14 (13)
0.7	14 (17)	13 (17)	15 (20)	9 (12)	10 (13)	11 (14)	9 (11)	10 (11)	10 (12)
0.8	11 (13)	11 (12)	12 (14)	8 (11)	8 (12)	9 (13)	9 (12)	9 (12)	10 (13)
0.9	24 (35)	21 (34)	20 (30)	12 (19)	11 (16)	11 (17)	8 (10)	8 (11)	9 (12)

Numbers in brackets are the estimation errors’ standard deviation

**Table 6:** Mean squared error ($\times 10^3$) for irregularly spaced fractional Gaussian noise featuring different degrees of missing observations for a range of Hurst parameters for the LoMPE estimation procedure

*H*	$n=256$			$n=512$			$n=1024$		
	Missingness proportion, *p*			Missingness proportion, *p*			Missingness proportion, *p*		
	5 %	10 %	20 %	5 %	10 %	20 %	5 %	10 %	20 %
0.6	2 (2)	2 (2)	3 (4)	1 (1)	1 (1)	1 (2)	1 (1)	1 (1)	1 (1)
0.7	3 (3)	3 (3)	3 (4)	2 (2)	2 (2)	2 (2)	2 (2)	2 (2)	3 (3)
0.8	3 (4)	3 (4)	4 (6)	3 (3)	3 (3)	4 (4)	3 (2)	4 (3)	5 (3)
0.9	3 (5)	4 (6)	4 (7)	3 (3)	4 (3)	4 (4)	4 (3)	5 (3)	6 (4)

Numbers in brackets are the estimation errors’ standard deviation

**Table 7:** Mean squared error ($\times 10^3$) for irregularly spaced fractionally integrated processes featuring different degrees of missing observations for a range of Hurst parameters, $H=d+1/2$, for the LoMPE estimation procedure

*H*	$n=256$			$n=512$			$n=1024$		
	Proportion of missingness, *p*			Proportion of missingness, *p*			Proportion of missingness, *p*		
	5 %	10 %	20 %	5 %	10 %	20 %	5 %	10 %	20 %
0.6	2 (3)	3 (4)	3 (5)	2 (2)	2 (2)	2 (2)	2 (1)	2 (1)	2 (1)
0.7	4 (5)	5 (6)	5 (5)	5 (4)	5 (4)	6 (4)	4 (3)	5 (3)	5 (4)
0.8	8 (9)	8 (9)	9 (9)	7 (6)	8 (6)	9 (7)	8 (5)	8 (5)	9 (6)
0.9	8 (8)	9 (10)	10 (10)	9 (7)	10 (8)	11 (10)	10 (6)	10 (6)	12 (7)

Numbers in brackets are the estimation errors’ standard deviation

Overall, our LoMPE method performs well when compared to methods that were specifically designed for regularly sampled series. LoMPE outperforms its competitors in over 75 % of cases and for three-quarters of those the improvement is greater than 40 %. Our method is slightly worse than Peng’s method for fractionally integrated series shown in Table 4, but mostly still better than the *wavelet* method for larger sample sizes.

These results are particularly pleasing since even though our method is designed for irregularly spaced data, it performs extremely well for regularly spaced time series.

### Performance for irregularly sampled data

Tables 5, 6 and 7 report the mean squared error for our LoMPE estimator on irregularly sampled time series for different degrees of missingness (up to 20 %). The tables show that higher degrees of missingness result in a slightly worse performance of the estimator; however, this decrease is small considering the irregular nature of the series, and the results are for the most part comparable with the results for the regular series. The supplementary material exhibits similar simulation results when we changed the missingness pattern from ‘missing at random’ to contiguous missing stretches in the manner of Junger and Ponce de Leon (2015). This shows a degree of robustness to different patterns of missingness.

**Table 8:** Empirical estimator bias $(\times 100)$ after aggregating fractional Brownian motion series ($n=512$) for a range of Hurst parameters featuring different degrees of missing observations to sampling intervals of size $\delta =2$ for three estimation methods

H	LoMPE			Peng			Wavelet		
	5 %	10 %	20 %	5 %	10 %	20 %	5 %	10 %	20 %
0.6	$-8$ (7)	$-8$ (8)	$-8$ (8)	$-10$ (13)	$-19$ (14)	$-37$ (15)	$-11$ (19)	$-26$ (20)	$-39$ (16)
0.7	$-6$ (7)	$-7$ (7)	$-7$ (7)	$-13$ (13)	$-25$ (15)	$-47$ (17)	$-12$ (22)	$-29$ (25)	$-45$ (19)
0.8	$$-3$$ (9)	$$-3$$ (8)	$$-4$$ (9)	$$-17$$ (15)	$$-32$$ (18)	$$ -61$$ (20)	$$-21$$ (27)	$$-41$$ (25)	$$-50$$ (21)
0.9	2 (11)	1 (11)	1 (11)	$-18$ (18)	$-42$ (20)	$-75$ (20)	$-22$ (28)	$-52$ (26)	$-58$ (18)

Numbers in brackets are the estimation errors’ standard deviation

**Fig. 5:**
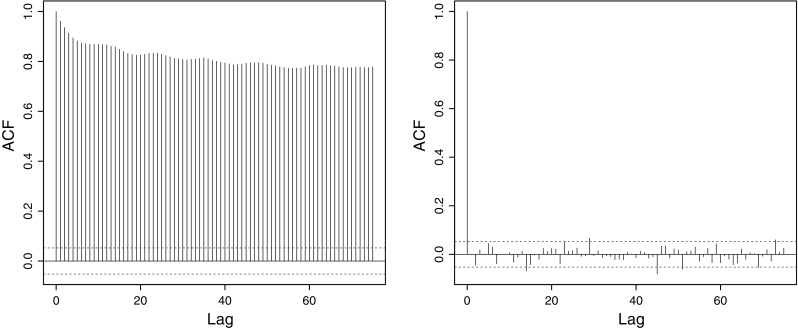
*Left* autocorrelation for the isotope series from Fig. 1 (treated as regularly spaced). *Right* autocorrelation for the LOCAAT-lifted isotope series

We also studied the empirical bias of our estimator. For reasons of brevity we do not report these bias results here, but the simulations can be found in Appendix C in the supplementary material. The results show that our method is competitive, achieving better results in over 65 % of cases and only slightly worse in the rest. As for the mean squared error results above, performance degrades for increasing missingness but still the results are remarkably good even when 20 % of observations are missing, and our proposed method is robust even at a significant loss of 40 % missing information (as detailed in the supplementary material). Indeed, in some cases the results are still competitive with those for the regular case in the previous section.

### Aggregation effects

We mentioned earlier that temporal aggregation is often used to mitigate the lack of regularly spaced samples. Several authors such as Granger and Joyeux (1980) and Beran et al. (2013) point out that aggregation over multiple time series can in itself *induce* long memory in the newly obtained process, even when the original process only had short-memory.

Motivated by this, we investigated the effect of temporal aggregation on long-memory processes via simulation. Specifically, we took regularly sampled long-memory processes (again fractional Brownian motion, fractional Gaussian noise and fractionally integrated classes) and induced an irregular sampling structure by randomly removing a percentage of the observations. We then aggregated (averaged) the observations in consecutive windows of length $\delta$ to mimic aggregation of irregularly observed time series, as usually done in practice. The long-memory intensity was estimated using our LoMPE method on the irregular data (no processing involved) and the Peng and wavelet methods on the aggregated sets. Table 8 shows the empirical bias for each procedure for a range of generating Hurst exponents and degree of missingness.

The results show that our direct LoMPE method produces dramatically better empirical bias results across most combinations of experimental conditions. For example, even for 5 % missingness, which shows the most conservative improvements, the median reduction in bias is four times that exhibited by the Peng and wavelet methods. The supplementary material shows similar results using fractional Gaussian noise and fractionally integrated processes with different degrees of aggregation, and also shows that the estimator variability increases markedly with increased aggregation span $\delta$.

*Estimation in the presence of a trend* Just as for classical wavelet methods, simulation experience has shown that our lifting-based method is not adversely affected by smooth trends, provided we use appropriately sized neighbourhoods to tune the number of wavelet vanishing moments. This is in contrast with other estimation methods, e.g. the local Whittle estimator, which are heavily affected by trends, to the point of becoming unusable (Abry et al. 2000).

## LoMPE analysis of environmental and climate science data

### Isotope ice core data

The sample autocorrelation of the isotope time series introduced in Sect. 1 is shown in the left panel of Fig. 5 and the autocorrelation of the LOCAAT-lifted series in the right panel, in both cases treating them as regularly spaced. The powerful decorrelation ability of lifting is clear.

**Fig. 6:**
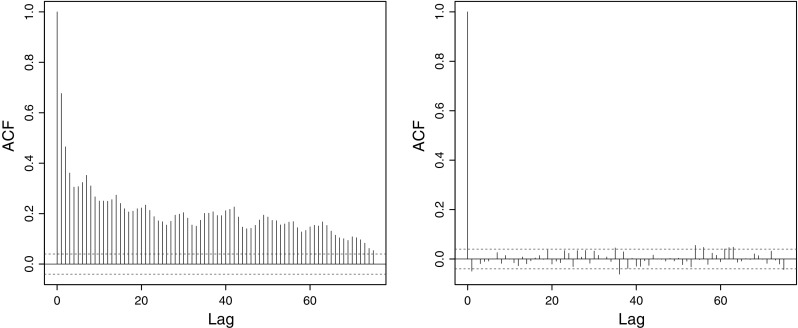
*Left* autocorrelation of the Bristol Ozone concentration series from Fig. 2 treated without missingness. *Right* autocorrelation after LOCAAT transformation

**Table 9:** Hurst parameter estimates for Ozone irregularly spaced time series for six British locations for the Windsor and Toumi (2001) method (W&T) and our proposed method (LoMPE)

	Bristol	Edinburgh	Leeds	London	Lough Navar	Rochester
W&T	0.700	0.760	0.755	0.780	0.755	0.778
LoMPE	0.847	0.804	0.827	0.832	0.837	0.851

Our LoMPE method estimates the Hurst parameter to be $\hat{H}= 0.76$ which indicates long memory, with an approximate bootstrap confidence interval of [0.7, 0.82]. Blender et al. (2006) reported a Hurst exponent of $\hat{H}=0.84$. In view of the demonstrated accuracy of our methods above, we would suggest that the literature is currently overestimating this parameter and hence the persistence of the isotope over long periods of time. This in turn leads to model miscalibration and inaccurate past reconstruction, e.g. greenhouse gases, and overestimation of their long-term effect in coupled ocean-atmosphere climate models (Fraedrich and Blender 2003; Wolff 2005; Blender et al. 2006).

Although the focus here has been Hurst estimation on ice-volume stratigraphy, many of these series’ characteristics—such as irregular time sampling—are common to many other paleoclimatic series. We have also applied our methodology to *electrical conductance* ice core series and argue that our estimation of the long-memory parameter for these series is more reliable than that in the literature. For reasons of brevity we do not include results here, but refer the reader to Appendix D in the supplementary material.

Our technique could be naturally applied to other series that might exhibit sampling irregularity and/or missingness.

### Atmospheric pollutants data

The autocorrelation before and after LOCAAT-transformation for the Bristol Ozone series is shown in Fig. 6 and again the powerful decorrelation effect is clear. We were unable to discern the precise method for Hurst parameters estimation from irregular series in Windsor and Toumi (2001). However, we report the values from their Fig. 8 and our estimates in Table 9. On the basis of our LoMPE estimates, we concur with the conclusion in Windsor and Toumi (2001) that estimates are consistent across the six sites, indicating that pollution persistence is similar across rural and urban geographical locations. However, our *H* estimates are, in general, higher than those reported. This observation is significant as it suggests that ozone is a secondary pollutant which possesses a greater degree of persistence in the atmosphere than previously recognized. Also note that in particular for ozone measurements, more persistent behaviour results in more predictable series (Turcotte 1997; Rehman and Siddiqi 2009) and easier detection of trends (Vyushin et al. 2007).

## Discussion and further work

Hurst exponent estimation is a recurrent topic in many scientific applications, with significant implications for modelling and data analysis. One important aspect of real-world datasets is that their collection and monitoring are often not straightforward, leading to missingness, or to the use of proxies with naturally irregular sampling structures.

This article has (i) identified that naive adaption of existing long-memory parameter estimation methods gives rise to inaccurate estimators and (ii) created a new estimator, LoMPE, that works naturally in the irregular/missing domain giving excellent and accurate results on a comprehensive range of persistent processes as well as showing unexpected excellent performance in the regularly spaced setting.

Backed up by the evidence of LoMPE’s performance, our ice core analyses point towards an overestimation of the isotope persistence over long periods of time and unrealistically low reported errors for Hurst exponent estimates in the literature. Our analysis of the atmospheric time series underlines that long memory is present independent of geographic monitoring site. The results also indicate that ozone, as a secondary pollutant, has a higher degree of persistence than has been previously recognized, and thus has potentially greater long-term implications on population-level respiratory health. However, LoMPE is not just restricted to the climate data applications that stimulated it, but can also be used in other contexts where irregular sampling or missing data are common.

For the estimator proposed in this paper, we restricted our attention to LOCAAT algorithms using a small number of neighbours and linear *predict* lifting steps. Future work might investigate higher order prediction schemes and larger neighbourhoods; also, the use of *adaptive* lifting schemes, such as Nunes et al. (2006), might provide benefits arising from improved decorrelation. They would also have the advantage of removing the *a priori* choice of a wavelet basis for our estimator. Finally, the estimation methods introduced in this article could be naturally extended to higher dimensions using the Voronoi polygon or tree-based lifting transforms introduced in Jansen et al. (2009). In the climate science context, a novel spatial Hurst dependence estimation would allow for inclusion of the geographical location and be conducive to dynamic spatial modelling.

An interesting avenue for future research would be to consider the use of compressed sensing methods and the non-uniform Fourier transform, (Marvasti 2001) or the Lomb-Scargle method to estimate the spectrum and thence the Hurst parameter.

### Electronic supplementary material

Below is the link to the electronic supplementary material.

Supplementary material 1 (pdf 137 KB)

## Figures and Tables

**Fig. 7:**
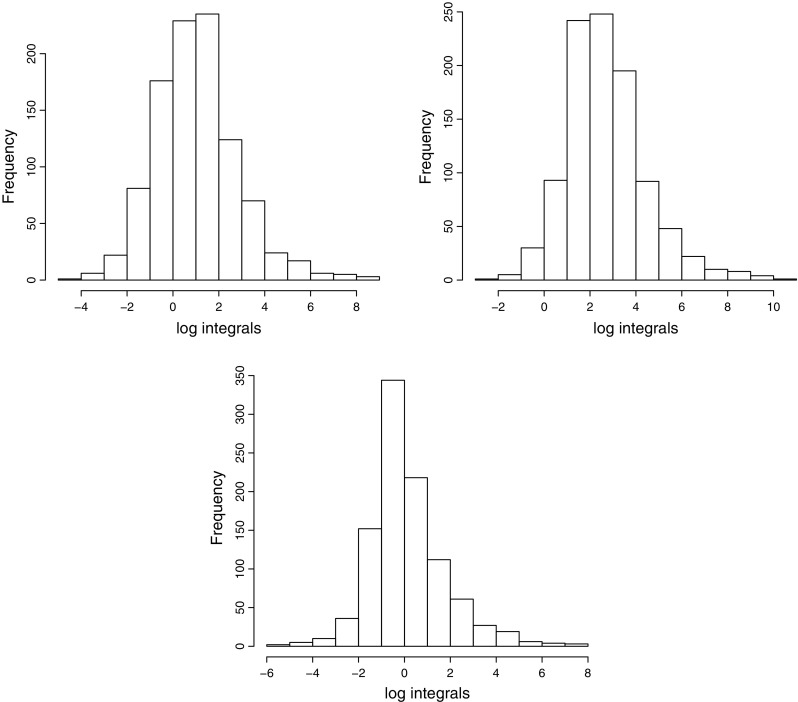
Three different interpoint distance samples (time sampling configurations) from Exp(1), $\chi ^2_3$ and U[0,1] distributions, with associated $\log _2$ integrals. The ($\log _2$) integrals are approximately normally distributed

**Fig. 8:**
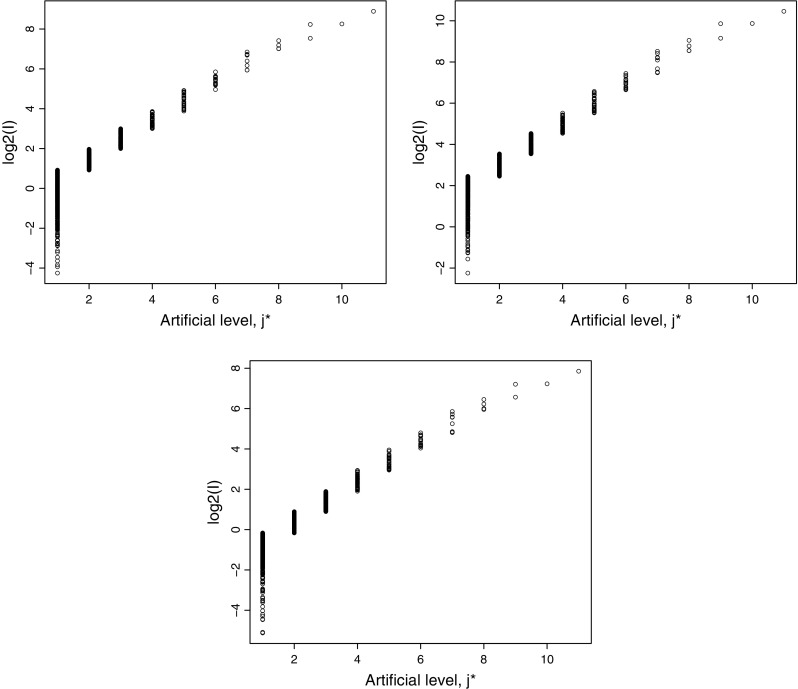
Three different interpoint distance samples (time sampling configurations) from an Exp(1), $\chi ^2_3$ and U[0,1] distributions, with associated $\log _2$ integrals, split into artificial levels, $j^{\star }$. There is a clear linear relationship between the $\log _2$ integrals and the artificial levels

## Proofs and theoretical results

This appendix gives the theoretical justification of the results from Sects. 3.1 and 4, following the notation outlined in the text.

### Proof of Proposition 1

Let $\{X_t\}$ be a zero-mean stationary long-memory series with autocovariance $\gamma _X(\tau ) \sim c_{\gamma } \tau ^{-\beta }$ with $\beta \in (0,1)$.

The autocovariance of $\{X_t\}$ can be written as ${\text {Cov}}(X_{t_i},X_{t_j})=\gamma _{X}(t_i-t_j) = {\mathbb {E}}(X_{t_i}X_{t_j})$, assuming ${\mathbb {E}}(X_t) = 0$. Hence, ${\mathbb {E}}(d_j) = 0$ and

12 $$\begin{aligned}&{\text {Cov}}( d_{j_r}, d_{j_k} ) = {\mathbb {E}}(d_{j_r} d_{j_k})\nonumber \\&\quad =\int _{{\mathbb {R}}}\tilde{\psi }_{j_r}(t)\left\{ \int _{{\mathbb {R}}}\tilde{\psi }_{j_k}(s) \gamma _{X}(t-s)\, ds\right\} \, dt, \end{aligned}$$

where $d_{j_r}=<X,\tilde{\psi }_{j_r}>$ for distinct times $j_r$ and $j_k$. Denote the interval length (i.e. continuous scale) of detail $d_{j_r}$ by $I_{r,j_r}$.

Since from  (6), the (dual) wavelet functions are linear combinations of scaling functions, Eq. (12) can be re-written as

13 $$\begin{aligned}&{\mathbb {E}}(d_{j_r}d_{j_k})=\int _{{\mathbb {R}}} \left\{ \tilde{\varphi }_{r,j_r}(t)-\sum _{i\in {{\mathscr {I}}_r}} a_i^r\tilde{\varphi }_{r,i}(t) \right\} \nonumber \\&\qquad \quad \times \int _{{\mathbb {R}}}\left\{ \tilde{\varphi }_{k,j_k}(s)-\sum _{j\in {{\mathscr {I}}_k}} a_j^k\tilde{\varphi }_{k,j}(s) \right\} \gamma _{X}(t-s)\ ds\ dt.\nonumber \\ \end{aligned}$$

As LOCAAT progresses, the (dual) scaling functions are defined recursively as linear combinations of (dual) scaling functions at the previous stage, from Eqs. (4) and (5).

By recursion the scaling functions in the above equation can be written as linear combinations of scaling functions at the first stage (i.e. $r=n$). Due to the linearity of the integral operator, (13) can be written as a linear combination of terms like

14 $$\begin{aligned} B_{n, i, j}:= & {} \int _{{\mathbb {R}}}\tilde{\varphi }_{n,i}(t)\left\{ \int _{{\mathbb {R}}}\tilde{\varphi }_{n,j}(s) \gamma _{X}(t-s)\,ds\right\} dt\nonumber \\= & {} \int _{{\mathbb {R}}}\tilde{\varphi }_{n,i}(t)\left( \tilde{\varphi }_{n,j}\star \gamma _{X} \right) (t)\, dt, \end{aligned}$$

where $\star$ is the convolution operator, and *i* and *j* refer to time locations that were involved in obtaining $d_{j_r}$ and $d_{j_k}$. Recall from Sect. 2.3 that the (dual) scaling functions are initially defined (at stage $r=n$) as scaled characteristic functions of the intervals associated with the observed times, i.e. $\tilde{\varphi }_{n,i}(t)=I^{-1}_{n,i}\chi _{I_{n,i}}(t)$ (Jansen et al. 2009). Using Parseval’s theorem in Eq.  (14) gives

15 $$\begin{aligned} B_{n, i, j}= & {} (2\pi )^{-1}\int _{{\mathbb {R}}} \hat{\tilde{\varphi }}_{n,i}(\omega ) \left( \overline{ \widehat{ \tilde{\varphi }_{n,j} \star \gamma _{X} } }\right) (\omega )\, d\omega \nonumber \\= & {} (2\pi )^{-1}\int _{{\mathbb {R}}}\hat{\tilde{\varphi }}_{n,i}(\omega ) \overline{\hat{\tilde{\varphi }}_{n,j}}(\omega ) \overline{f_{X}}(\omega )\, d\omega , \end{aligned}$$

where $\hat{f}$ is the Fourier transform of *f*. As the Fourier transform of an initial (dual) scaling function (scaled characteristic function on an interval, $(b-a)^{-1}\chi _{[a,b]}$) is

$$\begin{aligned}&\widehat{\left\{ (b-a)^{-1}\chi _{[a,b]}\right\} }(\omega )\\&\quad ={\text {sinc}}\left\{ \omega (b-a)/2 \right\} \exp \left\{ -\mathrm {i}\,\omega (b+a)/2 \right\} , \end{aligned}$$

where ${\text {sinc}}(x)=x^{-1}\sin (x)$ for $x\ne 0$ and ${\text {sinc}}(0) = 1$ is the (unnormalized) ${\text {sinc}}$ function, we can write (15) as

16 $$\begin{aligned}&\int _{{\mathbb {R}}} {\text {sinc}}\left( \omega I_{n,i}/2\right) {\text {sinc}}\left( \omega I_{n,j}/2\right) \exp \left\{ -\mathrm {i}\,\omega \delta (I_{n,i},I_{n,j}) \right\} \nonumber \\&\quad f_X(\omega ) d\omega , \end{aligned}$$

where $\delta (I_{n,i},I_{n,j})$ is the distance between the midpoints of intervals $I_{n,i}$ and $I_{n,j}$ at the initial stage *n*. Equation (16) can be interpreted as the Fourier transform of $u(x)=f_X(x) {\text {sinc}}\left( x I_{n,i}/2\right) {\text {sinc}}\left( x I_{n,j}/2\right)$ evaluated at $\delta (I_{n,i},I_{n,j})$.

Since the sinc function is infinitely differentiable and the spectrum is Lipschitz continuous, results on the decay properties of Fourier transforms (Shibata and Shimizu 2001, Theorem 2.2) imply that, for $i\ne j$, terms of the form $B_{n, i, j}$ decay as $O \left\{ \delta (I_{n,i},I_{n,j})^{-1} \right\}$. Hence the further away the time points are, the less autocorrelation is present in the wavelet domain and the rate of autocorrelation decay for the wavelet coefficients is of reciprocal order, thus faster than that of the original process.

### Proof of Proposition 2

As ${\text {Cov}}(X_{t_i},X_{t_j})=\gamma _{X}(t_i-t_j)$ and $d_{j_r}=<X,\tilde{\psi }_{j_r}>$, it follows that $d_{j_r}$ has mean zero (as the original process is zero-mean) and in a similar manner to (12) we have

17 $$\begin{aligned} {\text {Var}}(d_{j_r})\!=\!{\mathbb {E}}(d_{j_r}^2)\!=\!\int _{{\mathbb {R}}}\tilde{\psi }_{j_r}(t)\left\{ \int _{{\mathbb {R}}}\tilde{\psi }_{j_r}(s) \gamma _{X}(t\!-\!s)\ ds\right\} \ dt. \end{aligned}$$

As before, we denote the associated interval length of the detail $d_{j_r}$ by $I_{r,j_r}$.

Using the recursiveness in the dual wavelet construction (Eq. 6), it follows that the (dual) wavelet functions are linear combinations of scaling functions and Eq. (17) can be re-written as

18

As in Proposition 1, using Parseval’s theorem we obtain

19

where recall that the hat notation denotes the Fourier transform of a function and $\delta (I_{r,i},I_{r,j})$ denotes the distance between the midpoints of intervals $I_{r,i}$ and $I_{r,j}$.

Due to the artificial level construction, the sequence of lifting integrals is approximately log-linear in the artificial level, i.e. for those points $j_r$ in the $j^\star$th artificial level, we have $\log _{2} \left( I_{r,j_r}\right) = j^{\star } + \Delta$ where $\Delta \in \{ -1 + \log _2 (\alpha _0), \log _2 (\alpha _0) \}$. Hence $I_{r,j_r} = R 2^{j^{\star }}$ for some constant $R > 0$. This follows from the fact that the artificial levels are defined as a dyadic rescaling of the time range of the type $(2^{j^{\star }-1}\alpha _0,2^{j^{\star }}\alpha _0]$, and the result follows as on a $\hbox {log}_2$ scale, the artificial scale intervals become $(j^{\star }-1+\log _2(\alpha _0),j^{\star }+\log _2(\alpha _0)]$. For an alternative justification when using a quantile-based approach for the artificial scale construction, the reader is directed to Appendix B in the supplementary material. Now suppose $i=j$ and both points belong to the $j^{\star }$th artificial level. In Eq. (19) we make a change of variable $\eta =\omega R 2^{j^{\star }}$ to obtain

20 $$\begin{aligned} B_{r, i, i}= & {} \int _{{\mathbb {R}}} {\text {sinc}}^2 (\eta /2) {f_{X}} (\eta / R 2^{j^{\star }}) {\left( R 2^{j^{\star }}\right) }^{-1} d\eta \nonumber \\&\quad \sim \int _{{\mathbb {R}}} {\text {sinc}}^2 (\eta /2 ) c_{f}|\eta |^{-\alpha }{\left( R 2^{j^{\star }}\right) }^{\alpha -1} d\eta , \;(j^{\star } \rightarrow \infty ) \nonumber \\= & {} 2^{{j^{\star }}{(\alpha -1)}} \int _{{\mathbb {R}}} c_f R^{\alpha -1} {\text {sinc}}^2 (\eta /2)|\eta |^{-\alpha } d\eta \nonumber \\= & {} 2^{j^{\star } (\alpha -1)} R^{\alpha -1} 4 c_f \Gamma (-1-\alpha ) \sin (\pi \alpha /2), \end{aligned}$$

where $\alpha \in (0,1)$ and $\Gamma$ is the Gamma function. If $i\ne j$ are points from the same neighbourhood ${\mathscr {I}}_r$ and both belong to the same artificial level $j^{\star }$, then their artificial scale measure will be the same. Performing the same change of variable as above, we obtain

21 $$\begin{aligned} B_{r, i, j}\sim & {} \int _{{\mathbb {R}}} {\text {sinc}}^2 ( \eta /2) e^{-\mathrm {i}\,\eta } {\left( R 2^{j^{\star }}\right) }^{-1}c_{f}|\eta |^{-\alpha }{\left( R 2^{j^{\star }}\right) }^{\alpha } d\eta ,\nonumber \\&\quad (j^{\star } \rightarrow \infty ) \nonumber \\= & {} 2^{{j^{\star }}{(\alpha -1)}} c_f R^{\alpha -1} \int _{{\mathbb {R}}} {\text {sinc}}^2 (\eta /2)e^{-\mathrm {i}\,\eta }|\eta |^{-\alpha } d\eta \nonumber \\= & {} 2^{j^{\star } (\alpha -1)} R^{\alpha -1} 4 c_f (2^\alpha - 1) \sin (\pi \alpha /2) \Gamma (1-\alpha ).\nonumber \\ \end{aligned}$$

All terms in (18) involve points from the same neighbourhood ${\mathscr {I}}_r$, and thus using (20) and (21) together with the linearity of the integral operator, we have that

$$\begin{aligned} {\text {Var}}(d_{j_r}) \sim C \, 2^{j^{\star } (\alpha -1)}, \end{aligned}$$

where *C* is a constant depending on $c_f, R$ and $\alpha$. $\square$

## Establishing approximate log-linearity of the lifting integral

This appendix demonstrates the log-linearity of the lifting integral $I_{r,j_r}$ as a function of the artificial scale ($j^{\star }$ in the notation of Sect. 4), when the construction we follow is, by defining the artificial levels as inter-quantile intervals of the lifting integrals, segmenting at e.g. median, the 75 % percentile, the 87.5 % percentile and so on.

In what follows, we assume that the time locations $(t_i)_{i=0}^{N-1}$ corresponding to the long-range dependent (LRD) process $\{X_{t_i}\}_{i=0}^{N-1}$ are generated from a random sampling process. Recall that initially, the lifting algorithm works by constructing an initial set of ‘integrals’ associated to each observation $(t_i,X_i)$. One way of doing this is to construct intervals having the endpoints as the midpoints between the initial grid points $(t_i)_{i=0}^{N-1}$, see Nunes et al. (2006) and Jansen et al. (2009) for more details. We assume that $\log _{2}\left( I_{r,j_r}\right)$ follow a normal distribution with some mean $\mu$ and variance $\sigma ^2$. This assumption is realistic considering the additive nature of the update stage for integrals, and is also backed up by numerical simulations.

### Lemma 1

For simplicity of notation, let $I\,{:=}\,I_{r,j_r}$ be the random variable defined by the integral associated to the *r*th lifted observation, and assume that this was classified in the *j*th artificial level based on its size. Then

22 $$\begin{aligned} {\mathbb {E}}(\log _2 (I) \mid I \in j \text {th artificial level}) \approx j*a + k, \end{aligned}$$

i.e. the lifting integral associated with detail $d_{r,j_r}$ is approximately a log-linear function of its artificial level *j*.

### Proof

We start by noting that as the cumulative distribution function of a standard normal distribution, $\Phi (\cdot )$ and $\log _2(\cdot )$ are both non-decreasing functions, the integral classification using quantiles is equivalent to a classification of $\log _2(I)$ using the quantiles of the normal distribution (or, in practice, the sample quantiles).

Therefore, our problem is to compute the expectation of a normal random variable, say $Z=\log _2(I)$, conditional on it taking values on an inter-quantile interval of the $N(\mu , \sigma ^2)$ distribution, which we shall denote by $( z_{j-1}, z_{j}]$. Here $z_j=\mu +\sigma \Phi ^{-1}\left( 1 - 2^{-j} \right)$, reflecting our construction of artificial levels. Hence this random variable follows a truncated normal distribution, whose mean is given by

$$\begin{aligned}&{\mathbb {E}}(Z \mid Z\in (z_{j-1},z_{j}])\\&=\mu + \sigma \left\{ \Phi \left( \frac{z_{j}-\mu }{\sigma } \right) - \Phi \left( \frac{z_{j-1}-\mu }{\sigma } \right) \right\} ^{-1}\\&\quad \times \left\{ \phi \left( \frac{z_{j-1}-\mu }{\sigma } \right) -\phi \left( \frac{z_{j}-\mu }{\sigma } \right) \right\} \\&\approx \mu + \frac{\sigma ^2}{z_{j}-z_{j-1}} \left\{ 1- {\phi \left( \frac{z_{j-1}-\mu }{\sigma } \right) }^{-1}{\phi \left( \frac{z_{j}-\mu }{\sigma } \right) } \right\} \\&\text { using a Taylor expansion of } \Phi \text { and } \Phi ^\prime =\phi \\&\approx \mu + \frac{\sigma ^2}{z_{j}-z_{j-1}} \frac{(z_{j}-z_{j-1})(z_{j}+z_{j-1}-2\mu )}{2 \sigma ^2} \\&\text { using the expression of } \phi \text { and } e^x \approx 1+x\\&\approx \tfrac{1}{2} (z_{j}+z_{j-1}), \end{aligned}$$

where $\phi (x)$ denotes, as usual, the standard normal density.

Using a simple interpolation argument along the cumulative distribution function $\Phi$ and the definition $z_j=\mu +\sigma \Phi ^{-1}\left( 1 - 2^{-j} \right)$, we further express the above as

$$\begin{aligned}&{\mathbb {E}}(Z \mid Z\in (z_{j-1},z_{j}]) \approx \mu +\sigma \Phi ^{-1}\left\{ 1 -3 \cdot 2^{-(j+1)} \right\} \\&\quad =\mu + \sigma \Phi ^{-1}\left\{ f(j) \right\} , \hbox { where } f(j)=1 -3 \cdot 2^{-(j+1)}, \end{aligned}$$

and we want to show that $\Phi ^{-1} \circ f(\cdot )$ is a linear function (in *j*). This is equivalent to showing that $f^{-1} \circ \Phi (\cdot )$ is a linear function (in $z_{\alpha }$), where $f^{-1}(\alpha )=-\log _2(3/2-\alpha )$ for some $\alpha \in (0,1)$. Using a $\log (1+x) \approx x$ approximation, together with the linearity of $\Phi (z_{\alpha })$ (in $z_{\alpha }$) over most of its domain, our result follows. $\square$

We conjecture that $a=1$ and back this claim by simulations, as shown in the following section. This is also in agreement with the other way of constructing the artificial levels, as explained under Sect. A.2 in Appendix A.

### Empirical demonstration of the log-linearity of $I_{r,j_r}$

In this section we demonstrate empirical evidence to demonstrate the log-linearity of the lifting integral. To show breadth of applicability, we simulate random vectors of length $n=1000$ from a number of distributions to represent instances of sampling interval processes $(t_i)_{i=0}^{N-1}$, for a fixed trajectory and process $\{X_{t_i}\}_{i=1}^{N-1}$. More specifically, we simulate a sampling regime from each of (a) a Exp(1); (b) a $\chi ^2_3$ and (c) a U[0,1] distribution. We then perform the lifting algorithm and examine the lifting integrals produced, on a $\log _2$ scale. Figure 7 shows histograms of the LOCAAT integrals $\log _2(I)$ for the three different distributions; there is evidence of the approximate normality of the log-transformed integrals.

Figure 8 shows the relationship between $\log _2(I)$ and its corresponding artificial level $j^{\star }$ for the three different distributions. In all cases, the relationship is strikingly linear as a function of the artificial level, $j^{\star }$; moreover, the relationships have approximate unit slopes, with the intercepts varying across the initial distributions.
