# Supplementary material for: A wavelet lifting approach to long-memory estimation
Source: Stat Comput. 2016 Sep 3;27(6):1453–71. doi: 10.1007/s11222-016-9698-2 (PMC6979511; doi:10.1007/s11222-016-9698-2)
Supplement: Supplementary file 1 — Supplementary material 1 (pdf 137 KB) [file 11222_2016_9698_MOESM1_ESM.pdf]

Supplementary Material for "A wavelet lifting approach to long-memory estimation"

Marina I. Knight
Department of Mathematics, University of York
Guy P. Nason
School of Mathematics, University of Bristol
Matthew A. Nunes
Department of Mathematics and Statistics, Lancaster University

August 10, 2016

C Simulated performance of the Hurst exponent estimator

In this appendix we give additional details of the simulation study used to assess the performance of our Hurst estimation technique for both regularly and irregularly sampled long-memory processes.

As examples of stationary long-range dependent processes, we chose fractionally Gaussian noise and fractionally integrated processes; whilst the results in Section 4 of the article hold for stationary long-memory processes, we have also tested our proposed estimation technique for fractional Brownian motion processes, as the classical example of a self-similar process. We simulated the series for a range of Hurst exponents $H = 0.6, \dots, 0.9$. The time series lengths used in this study were $n = 256$, 512, and 1024 points.

For each simulated process, $\underline{X}^k$, $k = 1, \dots, K = 100$, we perform the estimation procedure described in Section 4 to obtain an estimate $\hat{H}^k$ and compare this to the truth, H. We report the mean squared error defined by

$$\text{MSE} = K^{-1} \sum_{k=1}^{K} (H - \hat{H}^k)^2, \tag{C.1}$$

as well as the bias incurred by the average estimate for the Hurst exponent over the K simulated series, in other words,

$$\text{Bias}(\bar{\hat{H}}) = \bar{\hat{H}} - H = K^{-1} \sum_{k=1}^{K} \hat{H}^k - H. \tag{C.2}$$

The estimation procedure described in Section 4 was implemented using an average of $P = 50$ estimated parameters (one for each lifting trajectory). In the following tables, our proposed method is denoted "LoMPE"; the simulations were repeated for the wavelet-based regression

technique of McCoy and Walden (1996); Jensen (1999), optimised for the choice of wavelet (denoted "*wavelet*"), as well as the residual variance method (Peng et al., 1994), which we denote "*Peng*".

The LoMPE method was implemented in *R* using modifications to the code contained in the *nlt* R package (Knight and Nunes, 2012). Both of the competitor estimation techniques are available within the *fArma* R package (Wuertz et al., 2013).

C.1 Regularly sampled time series

Table 1: Mean squared error ($\times 10^3$) for regularly spaced fractional Brownian motion series for a range of Hurst parameters for the estimation procedures described in the text. Numbers in brackets represent the standard deviation of estimation errors. Boxed numbers indicate best result.

	$n = 256$			$n = 512$			$n = 1024$		
H	Peng	Wavelet	LoMPE	Peng	Wavelet	LoMPE	Peng	Wavelet	LoMPE
0.6	19 (30)	29 (48)	$\boxed{12}$ (21)	13 (22)	20 (37)	$\boxed{9}$ (15)	$\boxed{10}$ (14)	13 (20)	$\boxed{10}$ (11)
0.7	25 (35)	34 (57)	$\boxed{12}$ (15)	14 (16)	21 (34)	$\boxed{8}$ (11)	9 (12)	15 (24)	$\boxed{8}$ (9)
0.8	19 (23)	24 (45)	$\boxed{11}$ (13)	13 (18)	17 (28)	$\boxed{7}$ (10)	12 (16)	15 (22)	$\boxed{8}$ (10)
0.9	$\boxed{23}$ (39)	34 (69)	28 (39)	15 (23)	17 (31)	$\boxed{13}$ (20)	12 (16)	16 (26)	$\boxed{7}$ (9)

Table 2: Mean squared error ($\times 10^3$) for regularly spaced fractional Gaussian noise for a range of Hurst parameters for the estimation procedures described in the text. Numbers in brackets represent the standard deviation of estimation errors. Boxed numbers indicate best result.

	$n = 256$			$n = 512$			$n = 1024$		
H	Peng	Wavelet	LoMPE	Peng	Wavelet	LoMPE	Peng	Wavelet	LoMPE
0.6	8 (11)	31 (50)	$\boxed{2}$ (2)	4 (6)	11 (19)	$\boxed{1}$ (1)	2 (3)	8 (13)	$\boxed{1}$ (1)
0.7	7 (8)	27 (49)	$\boxed{2}$ (3)	3 (5)	12 (19)	$\boxed{1}$ (1)	3 (3)	9 (15)	$\boxed{1}$ (1)
0.8	7 (11)	29 (70)	$\boxed{2}$ (3)	5 (6)	16 (26)	$\boxed{2}$ (3)	4 (6)	10 (16)	$\boxed{3}$ (2)
0.9	10 (13)	28 (64)	$\boxed{3}$ (4)	4 (5)	11 (15)	$\boxed{2}$ (3)	$\boxed{3}$ (5)	10 (17)	4 (2)

Table 3: Mean squared error ($\times 10^3$) for regularly spaced fractionally integrated series for a range of Hurst parameters, $H = d + 1/2$, for the estimation procedures described in the text. Numbers in brackets represent the standard deviation of estimation errors. Boxed numbers indicate best result.

	$n = 256$			$n = 512$			$n = 1024$		
H	Peng	Wavelet	LoMPE	Peng	Wavelet	LoMPE	Peng	Wavelet	LoMPE
0.6	8 (9)	25 (39)	$\boxed{3}$ (4)	4 (6)	16 (39)	$\boxed{1}$ (2)	2 (2)	8 (13)	$\boxed{1}$ (1)
0.7	8 (11)	29 (39)	$\boxed{4}$ (5)	$\boxed{4}$ (5)	9 (15)	$\boxed{4}$ (4)	$\boxed{3}$ (3)	6 (10)	4 (3)
0.8	11 (16)	28 (39)	$\boxed{6}$ (8)	7 (8)	18 (34)	$\boxed{6}$ (5)	$\boxed{4}$ (5)	6 (11)	6 (4)
0.9	12 (15)	30 (53)	$\boxed{7}$ (8)	$\boxed{7}$ (10)	11 (18)	8 (7)	$\boxed{4}$ (6)	8 (14)	9 (5)

Tables 1, 2 and 3 (resp. Tables 4, 5 and 6) show our simulation results for the range of Hurst parameters $H = 0.6, \ldots, 0.9$ across all three methods in terms of mean square error and bias. Overall, our proposed estimation method is competitive with traditional methods, and in particular outperforms the other methods for the nonstationary fractional Brownian motion series.

Table 4: Empirical bias ($\times 100$) of Hurst estimation on fractional Brownian motion realizations. Numbers in brackets are the estimation errors' standard deviation. Boxed numbers indicate best result.

	$n = 256$			$n = 512$			$n = 1024$		
H	Peng	Wavelet	LoMPE	Peng	Wavelet	LoMPE	Peng	Wavelet	LoMPE
0.6	−8 (10)	−8 (15)	$\boxed{-4}$ (10)	$\boxed{-5}$ (10)	−7 (12)	−6 (7)	$\boxed{-5}$ (8)	−5 (10)	−8 (6)
0.7	−10 (12)	−10 (15)	$\boxed{-1}$ (11)	−7 (9)	−8 (12)	$\boxed{-5}$ (7)	$\boxed{-5}$ (8)	−6 (11)	−6 (6)
0.8	−9 (11)	−9 (13)	$\boxed{4}$ (10)	−8 (9)	−8 (11)	$\boxed{-1}$ (8)	−7 (9)	−7 (10)	$\boxed{-5}$ (8)
0.9	$\boxed{-10}$ (11)	−11 (15)	$\boxed{10}$ (13)	−8 (9)	−8 (10)	$\boxed{4}$ (11)	−8 (8)	−8 (10)	$\boxed{-1}$ (8)

Table 5: Empirical bias ($\times 100$) of Hurst estimation on fractional Gaussian noise realizations. Numbers in brackets are the estimation errors' standard deviation. Boxed numbers indicate best result.

	$n = 256$			$n = 512$			$n = 1024$		
H	Peng	Wavelet	LoMPE	Peng	Wavelet	LoMPE	Peng	Wavelet	LoMPE
0.6	−4 (8)	−10 (15)	$\boxed{-1}$ (4)	−2 (6)	−3 (10)	$\boxed{-1}$ (3)	$\boxed{-2}$ (4)	−4 (8)	$\boxed{-2}$ (2)
0.7	−3 (8)	−5 (16)	$\boxed{-2}$ (4)	−3 (5)	−5 (10)	$\boxed{-2}$ (3)	$\boxed{-2}$ (5)	−4 (9)	−3 (2)
0.8	−3 (8)	−6 (16)	$\boxed{-2}$ (5)	$\boxed{-3}$ (6)	−6 (11)	−4 (3)	$\boxed{-3}$ (6)	−4 (9)	−5 (2)
0.9	−4 (9)	−6 (16)	$\boxed{-1}$ (5)	$\boxed{-3}$ (6)	−4 (10)	−4 (3)	$\boxed{-3}$ (5)	−4 (9)	−6 (2)

Table 6: Empirical bias ($\times 100$) of Hurst estimation on fractionally integrated process realizations. Numbers in brackets are the estimation errors' standard deviation. Boxed numbers indicate best result.

	$n = 256$			$n = 512$			$n = 1024$		
H	Peng	Wavelet	LoMPE	Peng	Wavelet	LoMPE	Peng	Wavelet	LoMPE
0.6	−4 (8)	−5 (12)	$\boxed{-2}$ (5)	−3 (6)	−6 (12)	$\boxed{-3}$ (3)	$\boxed{-2}$ (3)	−5 (8)	−3 (2)
0.7	−5 (8)	−8 (17)	$\boxed{-4}$ (5)	$\boxed{-4}$ (5)	−7 (10)	−6 (3)	$\boxed{-3}$ (4)	−5 (7)	−6 (2)
0.8	$\boxed{-6}$ (9)	−9 (18)	−6 (6)	$\boxed{-5}$ (7)	−6 (11)	−7 (4)	$\boxed{-4}$ (5)	−5 (9)	−8 (3)
0.9	$\boxed{-6}$ (9)	−6 (14)	−6 (6)	$\boxed{-5}$ (7)	−7 (13)	−8 (4)	$\boxed{-4}$ (5)	$\boxed{-4}$ (7)	−9 (3)

C.2 Irregularly sampled time series

To evaluate the LoMPE Hurst parameter estimation technique for irregular time series, we performed an additional study. After simulating time series with different long memory properties as above, we induced an irregular structure by randomly removing a portion of observations. More specifically, for each value of $H = 0.6, \dots, 0.9$, we created irregular time series with increasing proportions of missingness, namely $p = 5\%, 10\%, 15\%, 20\%$. We then computed the average error and bias as before (see equations (C.1) and (C.2)), by performing our proposed LoMPE estimation method on the irregular time series.

Table 7: Mean squared error ($\times 10^3$) for irregularly spaced fractional Brownian motion series featuring different degrees of missing observations for a range of Hurst parameters for the LoMPE estimation procedure. Numbers in brackets are the estimation errors' standard deviation.

	$n = 256$				$n = 512$				$n = 1024$			
	Proportion of missingness, p				Proportion of missingness, p				Proportion of missingness, p			
H	5%	10%	15%	20%	5%	10%	15%	20%	5%	10%	15%	20%
0.6	13 (22)	14 (23)	15 (25)	16 (25)	11 (16)	12 (17)	12 (18)	13 (19)	12 (12)	13 (13)	13 (13)	14 (13)
0.7	14 (17)	13 (17)	14 (18)	15 (20)	9 (12)	10 (13)	10 (13)	11 (14)	9 (11)	10 (11)	10 (12)	10 (12)
0.8	11 (13)	11 (12)	11 (13)	12 (14)	8 (11)	8 (12)	8 (12)	9 (13)	9 (12)	9 (12)	10 (12)	10 (13)
0.9	24 (35)	21 (34)	22 (33)	20 (30)	12 (19)	11 (16)	11 (16)	11 (17)	8 (10)	8 (11)	9 (11)	9 (12)

Table 8: Mean squared error ($\times 10^3$) for irregularly spaced fractional Gaussian noise featuring different degrees of missing observations for a range of Hurst parameters for the LoMPE estimation procedure. Numbers in brackets are the estimation errors' standard deviation.

	$n = 256$				$n = 512$				$n = 1024$			
	Proportion of missingness, p				Proportion of missingness, p				Proportion of missingness, p			
H	5%	10%	15%	20%	5%	10%	15%	20%	5%	10%	15%	20%
0.6	2 (2)	2 (2)	2 (3)	3 (4)	1 (1)	1 (1)	1 (1)	1 (2)	1 (1)	1 (1)	1 (1)	1 (1)
0.7	3 (3)	3 (3)	3 (4)	3 (4)	2 (2)	2 (2)	2 (2)	2 (2)	2 (2)	2 (2)	3 (2)	3 (3)
0.8	3 (4)	3 (4)	3 (5)	4 (6)	3 (3)	3 (3)	4 (3)	4 (4)	3 (2)	4 (3)	4 (3)	5 (3)
0.9	3 (5)	4 (6)	4 (6)	4 (7)	3 (3)	4 (3)	4 (4)	4 (4)	4 (3)	5 (3)	6 (3)	6 (4)

Table 9: Mean squared error ($\times 10^3$) for irregularly spaced fractionally integrated processes featuring different degrees of missing observations for a range of Hurst parameters, $H = d + 1/2$, for the LoMPE estimation procedure. Numbers in brackets are the estimation errors' standard deviation.

	$n = 256$				$n = 512$				$n = 1024$			
	Proportion of missingness, p				Proportion of missingness, p				Proportion of missingness, p			
$H = d + \frac{1}{2}$	5%	10%	15%	20%	5%	10%	15%	20%	5%	10%	15%	20%
0.6	2 (3)	3 (4)	3 (4)	3 (5)	2 (2)	2 (2)	2 (2)	2 (2)	2 (1)	2 (1)	2 (1)	2 (1)
0.7	4 (5)	5 (6)	5 (6)	5 (5)	5 (4)	5 (4)	5 (4)	6 (4)	4 (3)	5 (3)	5 (4)	5 (4)
0.8	8 (9)	8 (9)	9 (10)	9 (9)	7 (6)	8 (6)	8 (7)	9 (7)	8 (5)	8 (5)	9 (6)	9 (6)
0.9	8 (8)	9 (10)	10 (10)	10 (10)	9 (7)	10 (8)	10 (8)	11 (10)	10 (6)	10 (6)	11 (7)	12 (7)

Table 10: Empirical bias (×100) of LoMPE estimation for fractional Brownian motion realizations with different degrees of missing observations. Numbers in brackets are the estimation errors' standard deviation.

	$n = 256$				$n = 512$				$n = 1024$			
	Proportion of missingness, p				Proportion of missingness, p				Proportion of missingness, p			
H	5%	10%	15%	20%	5%	10%	15%	20%	5%	10%	15%	20%
0.6	-5 (10)	-6 (10)	-6 (10)	-7 (10)	-8 (7)	-8 (8)	-8 (8)	-8 (8)	-9 (6)	-9 (6)	-10 (6)	-10 (6)
0.7	-2 (11)	-3 (11)	-4 (11)	-4 (12)	-6 (7)	-7 (7)	-7 (7)	-7 (7)	-8 (6)	-8 (6)	-8 (6)	-8 (7)
0.8	2 (10)	1 (10)	1 (11)	0 (11)	-3 (8)	-3 (8)	-4 (8)	-4 (9)	-6 (8)	-6 (8)	-6 (8)	-7 (8)
0.9	8 (13)	7 (13)	7 (13)	6 (13)	2 (11)	1 (10)	1 (11)	1 (11)	-2 (9)	-3 (9)	-3 (9)	-3 (9)

Table 11: Empirical bias (×100) of LoMPE estimation for fractional Gaussian noise realizations with different degrees of missing observations. Numbers in brackets are the estimation errors' standard deviation.

	$n = 256$				$n = 512$				$n = 1024$			
	Proportion of missingness, p				Proportion of missingness, p				Proportion of missingness, p			
H	5%	10%	15%	20%	5%	10%	15%	20%	5%	10%	15%	20%
0.6	-2 (4)	-2 (4)	-2 (4)	-2 (5)	-2 (3)	-2 (3)	-2 (3)	-2 (3)	-2 (2)	-2 (2)	-2 (2)	-3 (2)
0.7	-2 (5)	-3 (5)	-3 (4)	-3 (4)	-3 (3)	-3 (3)	-3 (3)	-4 (3)	-4 (2)	-4 (2)	-5 (2)	-5 (2)
0.8	-2 (5)	-3 (5)	-3 (5)	-4 (5)	-5 (3)	-5 (3)	-6 (3)	-6 (3)	-5 (2)	-6 (2)	-6 (2)	-6 (2)
0.9	-2 (5)	-3 (5)	-3 (6)	-4 (5)	-5 (3)	-5 (3)	-6 (3)	-6 (3)	-6 (2)	-7 (2)	-7 (2)	-7 (2)

Table 12: Empirical bias (×100) of LoMPE estimation for fractional Gaussian noise realizations with different degrees of missing observations. Numbers in brackets are the estimation errors' standard deviation.

	$n = 256$				$n = 512$				$n = 1024$			
	Proportion of missingness, p				Proportion of missingness, p				Proportion of missingness, p			
H	5%	10%	15%	20%	5%	10%	15%	20%	5%	10%	15%	20%
0.6	-2 (4)	-2 (5)	-2 (4)	-3 (5)	-3 (3)	-3 (3)	-3 (3)	-3 (3)	-4 (2)	-4 (2)	-4 (2)	-4 (2)
0.7	-4 (4)	-5 (5)	-5 (5)	-5 (4)	-6 (3)	-6 (3)	-7 (3)	-7 (3)	-6 (2)	-6 (2)	-7 (3)	-7 (2)
0.8	-7 (6)	-7 (6)	-7 (6)	-8 (5)	-8 (4)	-8 (4)	-8 (4)	-8 (4)	-8 (3)	-8 (3)	-9 (3)	-9 (3)
0.9	-7 (6)	-8 (6)	-8 (6)	-8 (6)	-9 (4)	-9 (4)	-9 (4)	-10 (5)	-9 (3)	-10 (3)	-10 (4)	-10 (4)

Tables 7, 8 and 9 (resp. Tables 10, 11 and 12) report the mean square error and bias for the irregularly sampled time series for the different proportions of missingness for varying Hurst exponent. The results for the irregular time series indicate that there is a slight decrease in performance as the amount of missingness increases. However, the results are quite consistent and are reasonable considering the increasing irregular structure. In fact, in some cases the estimation results are competitive with the results for the methods for the regular case above.

C.3 Performance investigation for other missingness scenarios

To evaluate the performance of our Hurst exponent estimator in additional scenarios involving data missingness, we have also repeated the simulation study described in Section C.2 for $n = 512$ in the case of 40%. This amount of missingness is considered as extreme for climatic series (Junger and Ponce de Leon, 2015). Whilst there is an understandable degradation in performance, our proposed method is robust even at such significant loss of information (Tables 13–15).

In addition, we have generated long memory processes with more "structured" missingness. Specifically, for series of length $n = 512$, we have induced missingness by removing 10 stretches of data, each stretch consisting of 10 contiguous observations. This missingness structure represents an overall missingness level of approximately 20%. Inducing this structured missingness is in a similar spirit to other studies in the literature (e.g. Junger and Ponce de Leon (2015)) to mimick recording malfunctions. Reassuringly, this structure does not overly degrade the performance of our estimator, with the MSE results being similar to those in Section C.2.

Both scenarios (40% missingness and 20% structured missingness) are reported in Tables 13–15 below. For comparison to the results for regular series (no missingness) and randomly generated missingness (Section C.2), these results are also reported in the tables.

Table 13: Mean squared error ($\times 10^3$) for irregularly spaced fractional Brownian motion series featuring different degrees and structure of missingness for a range of Hurst parameters for the LoMPE estimation procedure. Numbers in brackets are the estimation errors' standard deviation.

	Regular grid			Random missingness proportion, p				Structured missingness
H	Peng	Wavelet	LoMPE	5%	10%	20%	40%	20%
0.6	13 (22)	20 (37)	9 (15)	11 (16)	12 (17)	13 (19)	20 (25)	16 (21)
0.7	14 (16)	21 (34)	8 (11)	9 (12)	10 (13)	11 (14)	17 (20)	13 (15)
0.8	13 (18)	17 (28)	7 (10)	8 (11)	8 (12)	9 (13)	17 (19)	10 (15)
0.9	15 (23)	17 (31)	13 (20)	12 (19)	11 (16)	11 (17)	14 (19)	12 (17)

C.4 Aggregation effects

We also conducted a simulation study to assess the effects of aggregation on our Hurst estimation method. More specifically, in a similar manner to the simulations above, we took a number of regularly sampled long memory processes and induced an irregular sampling structure by removing a percentage of the observations. We then aggregated (summed) the observations in consecutive windows of length δ to mimic aggregation of irregularly observed time series into those with a regular sampling interval. We then performed various Hurst estimation procedures on these

Table 14: Mean squared error ($\times 10^3$) for irregularly spaced fractional Gaussian noise featuring different degrees and structure of missingness for a range of Hurst parameters for the LoMPE estimation procedure. Numbers in brackets are the estimation errors' standard deviation.

	Regular grid			Random missingness proportion, p				Structured missingness
H	Peng	Wavelet	LoMPE	5%	10%	20%	40%	20%
0.6	4 (6)	11 (19)	1 (1)	1 (1)	1 (1)	1 (2)	2 (2)	1 (2)
0.7	3 (5)	12 (19)	1 (1)	2 (2)	2 (2)	2 (2)	4 (4)	2 (3)
0.8	5 (6)	16 (26)	2 (3)	3 (3)	3 (3)	4 (4)	9 (7)	4 (4)
0.9	4 (5)	11 (15)	2 (3)	3 (3)	4 (3)	4 (4)	8 (6)	3 (4)

Table 15: Mean squared error ($\times 10^3$) for irregularly spaced fractionally integrated processes featuring different degrees and structure of missingness for a range of Hurst parameters, $H = d + 1/2$, for the LoMPE estimation procedure. Numbers in brackets are the estimation errors' standard deviation.

	Regular grid			Random missingness proportion, p				Structured missingness
H	Peng	Wavelet	LoMPE	5%	10%	20%	40%	20%
0.6	4 (6)	16 (39)	1 (2)	2 (2)	2 (2)	2 (2)	3 (3)	2 (3)
0.7	4 (5)	9 (15)	4 (4)	5 (4)	5 (4)	6 (4)	8 (6)	6 (6)
0.8	7 (8)	18 (34)	6 (5)	7 (6)	8 (6)	9 (7)	13 (9)	8 (7)
0.9	7 (10)	11 (18)	8 (7)	9 (7)	10 (8)	11 (10)	17 (13)	10 (9)

regularly-sampled series. Tables 16 – 18 show the estimation bias ($\times 10^2$) for the estimation procedures, for a range of generating Hurst exponents, degree of missingness and aggregation levels.

The tables show that in general, our LoMPE estimation method (performed on the irregular observations directly) shows less estimation bias compared with competitor methods for a range of long-memory processes, especially for Fractional Brownian motion series.

D Application of methodology to other environmental datasets

This appendix applies our proposed methodology from Section 4 to other data arising in climate science. More specifically, we study electrical conductance ice core series obtained from the World Data Center for Paleoclimatology in Boulder, USA (`http://www.ncdc.noaa.gov/paleo/icecore/`). Figure C.1 shows two series of lengths $n = 2145$ and $n = 3158$ from the European Project for Ice Coring in Antarctica (EPICA Dome C) ice core. These irregularly spaced series arise from two conductance experiments, namely the D/C conductance and dielectric profiling, see Augustin et al. (2004) for more details.

We applied our LoMPE Hurst estimation procedure to these two series to estimate their long-memory. Figure C.2 (left) shows the autocorrelation of the `D/C` and `dielectric` time series; the autocorrelation function of their LOCAAT-transformed versions shown in Figure C.2 (right) demonstrates significant decorrelation.

Our LoMPE method estimated the Hurst parameter for the `D/C` series to be $\hat{H} = 0.84$ (approx confidence interval [0.75,0.93]) and $\hat{H} = 0.78$ (approx confidence interval [0.71,0.85]) for the `dielectric` series. King (2005) estimated the parameters to be $\hat{H} = 0.795 \pm 0.013$ and

Table 16: Empirical estimator bias ($\times 100$) after aggregating fractional Brownian motion series for a range of Hurst parameters featuring different degrees of missing observations to sampling intervals of size δ for three estimation methods. Numbers in brackets are the estimation errors' standard deviation.

	LoMPE				Peng				Wavelet			
H	5%	10%	15%	20%	5%	10%	15%	20%	5%	10%	15%	20%
$n = 256, \delta = 2$												
0.6	-5 (10)	-6 (10)	-6 (10)	-7 (10)	-11 (15)	-17 (17)	-27 (17)	-38 (20)	-12 (23)	-17 (23)	-22 (26)	-27 (23)
0.7	-3 (11)	-3 (11)	-4 (11)	-4 (12)	-14 (16)	-26 (19)	-36 (21)	-48 (21)	-14 (28)	-24 (28)	-28 (26)	-30 (28)
0.8	2 (11)	1 (10)	1 (11)	0 (11)	-14 (16)	-26 (23)	-43 (23)	-57 (22)	-14 (32)	-25 (33)	-37 (27)	-41 (28)
0.9	8 (13)	7 (13)	7 (13)	6 (13)	-20 (21)	-33 (24)	-51 (26)	-71 (23)	-15 (31)	-33 (36)	-42 (30)	-49 (29)
$n = 512, \delta = 2$												
0.6	-8 (7)	-8 (8)	-8 (8)	-8 (8)	-10 (13)	-19 (14)	-28 (15)	-37 (15)	-11 (19)	-26 (20)	-32 (19)	-39 (16)
0.7	-6 (7)	-7 (7)	-7 (7)	-7 (7)	-13 (13)	-25 (15)	-35 (13)	-47 (17)	-12 (22)	-29 (26)	-43 (19)	-45 (19)
0.8	-3 (9)	-3 (8)	-4 (8)	-4 (9)	-17 (15)	-32 (18)	-50 (17)	-61 (20)	-21 (27)	-41 (25)	-54 (19)	-50 (21)
0.9	2 (11)	1 (11)	1 (11)	1 (11)	-18 (18)	-42 (20)	-62 (20)	-75 (20)	-22 (28)	-52 (26)	-58 (22)	-58 (18)
$n = 512, \delta = 4$												
0.6					-7 (17)	-7 (18)	-7 (18)	-9 (17)	-6 (25)	-6 (25)	-6 (26)	-10 (27)
0.7					-10 (14)	-10 (14)	-11 (15)	-13 (15)	-4 (27)	-5 (27)	-6 (26)	-9 (28)
0.8					-10 (13)	-10 (13)	-12 (15)	-12 (13)	-8 (27)	-8 (27)	-9 (28)	-10 (28)
0.9					-10 (14)	-10 (14)	-12 (16)	-13 (16)	-4 (25)	-4 (25)	-6 (27)	-8 (28)
$n = 1024, \delta = 2$												
0.6	-9 (6)	-9 (6)	-10 (6)	-10 (6)	-11 (11)	-22 (11)	-31 (12)	-40 (14)	-17 (19)	-30 (18)	-38 (15)	-42 (13)
0.7	-8 (6)	-8 (6)	-8 (6)	-8 (7)	-13 (13)	-32 (14)	-43 (15)	-53 (15)	-16 (23)	-41 (21)	-46 (16)	-49 (14)
0.8	-6 (8)	-6 (8)	-7 (8)	-7 (8)	-19 (14)	-40 (15)	-53 (16)	-66 (16)	-28 (24)	-55 (20)	-58 (16)	-55 (16)
0.9	-2 (9)	-3 (9)	-3 (9)	-3 (9)	-27 (18)	-52 (18)	-66 (17)	-79 (17)	-39 (31)	-66 (20)	-65 (17)	-62 (19)
$n = 1024, \delta = 4$												
0.6					-6 (14)	-6 (14)	-7 (14)	-8 (14)	-5 (18)	-6 (18)	-7 (19)	-10 (21)
0.7					-7 (13)	-7 (13)	-9 (13)	-12 (15)	-2 (15)	-3 (16)	-5 (17)	-9 (21)
0.8					-8 (12)	-9 (12)	-11 (14)	-15 (15)	-8 (17)	-9 (17)	-13 (20)	-20 (26)
0.9					-10 (11)	-11 (12)	-11 (12)	-17 (16)	-5 (18)	-6 (19)	-8 (22)	-16 (26)
$n = 1024, \delta = 8$												
0.6					-7 (17)	-7 (17)	-7 (17)	-7 (17)	-5 (26)	-5 (26)	-5 (26)	-5 (26)
0.7					-9 (16)	-9 (16)	-9 (16)	-9 (16)	-2 (24)	-2 (24)	-2 (24)	-2 (24)
0.8					-10 (15)	-10 (15)	-10 (15)	-10 (15)	-12 (26)	-12 (26)	-12 (26)	-12 (26)
0.9					-12 (14)	-12 (14)	-12 (14)	-12 (14)	-4 (25)	-4 (25)	-4 (25)	-4 (25)

$\hat{H} = 0.85 \pm 0.012$ respectively. However, it is not clear what the status of the $\pm$ errors is for these H estimates and, bearing in mind the standard errors observed in the simulations above (especially for empirical bias for even the established methods), it seems that the standard errors in the literature are perhaps too small.

References

Augustin, L., Barbante, C., Barnes, P. R. F., Barnola, J.-M., Bigler, M., Castellano, E., Cattani, O., Chappellaz, J., Dahl-Jensen, D., Delmonte, B., et al. (2004) Eight glacial cycles from an antarctic ice core, *Nature*, **429**, 623–628.

Jensen, M. J. (1999) Using wavelets to obtain a consistent ordinary least squares estimator of the long-memory parameter, *Journal of Forecasting*, **18**, 17–32.

Junger, W. L. and Ponce de Leon, A. (2015) Imputation of missing data in time series for air pollutants, *Atmospheric Environment*, **102**, 96–104.

Table 17: Empirical estimator bias $(\times 100)$ after aggregating fractional Gaussian noise for a range of Hurst parameters featuring different degrees of missing observations to sampling intervals of size δ for three estimation methods. Numbers in brackets are the estimation errors' standard deviation.

	LoMPE				Peng				Wavelet			
H	5%	10%	15%	20%	5%	10%	15%	20%	5%	10%	15%	20%
						$n = 256, \delta = 2$						
0.6	-2 (4)	-2 (4)	-2 (4)	-2 (5)	-6 (11)	-6 (11)	-7 (10)	-7 (10)	-5 (29)	-6 (27)	-9 (28)	-5 (26)
0.7	-2 (5)	-3 (5)	-3 (4)	-3 (5)	-4 (12)	-4 (12)	-5 (12)	-6 (12)	-10 (26)	-10 (25)	-9 (27)	-12 (28)
0.8	-2 (5)	-3 (5)	-3 (5)	-4 (5)	-4 (12)	-5 (11)	-5 (10)	-6 (11)	-1 (28)	-3 (27)	-2 (25)	-5 (28)
0.9	-3 (5)	-3 (5)	-3 (6)	-4 (5)	-5 (13)	-6 (13)	-7 (13)	-8 (13)	-7 (25)	-7 (25)	-9 (23)	-10 (25)
						$n = 512, \delta = 2$						
0.6	-2 (3)	-2 (3)	-2 (3)	-2 (3)	-3 (8)	-3 (8)	-4 (8)	-5 (9)	-5 (18)	-5 (17)	-6 (16)	-9 (18)
0.7	-3 (3)	-3 (3)	-3 (3)	-4 (3)	-4 (7)	-5 (7)	-5 (7)	-6 (7)	-5 (18)	-5 (19)	-5 (17)	-7 (17)
0.8	-5 (3)	-5 (3)	-6 (3)	-6 (3)	-5 (8)	-5 (8)	-6 (8)	-7 (8	-6 (19)	-7 (19)	-6 (18)	-9 (19)
0.9	-5 (3)	-5 (3)	-6 (3)	-6 (3)	-5 (8)	-6 (9)	-7 (8)	-8 (8)	-5 (15)	-5 (14)	-7 (13)	-8 (14)
						$n = 512, \delta = 4$						
0.6					-3 (10)	-3 (11)	-4 (11)	-5 (10)	-7 (29)	-6 (28)	-8 (26)	-9 (29)
0.7					-6 (10)	-6 (10)	-6 (10)	-7 (10)	-8 (29)	-9 (32)	-11 (30)	-9 (28)
0.8					-6 (11)	-6 (11)	-7 (12)	-8 (11)	-7 (29)	-8 (29)	-7 (27)	-11 (28)
0.9					-6 (12)	-6 (12)	-7 (12)	-7 (12)	-7 (25)	-7 (25)	-9 (24)	-9 (24)
						$n = 1024, \delta = 2$						
0.6	-2 (2)	-2 (2)	-2 (2)	-3 (2)	-3 (5)	-4 (5)	-4 (5)	-5 (6)	-5 (12)	-4 (12)	-6 (12)	-6 (12)
0.7	-4 (2)	-4 (2)	-5 (2)	-5 (2)	-3 (6)	-4 (6)	-4 (6)	-5 (6)	-2 (11)	-4 (11)	-4 (10)	-5 (11)
0.8	-5 (2)	-6 (2)	-6 (2)	-6 (3)	-5 (7)	-5 (7)	-6 (7)	-6 (7)	-4 (11)	-5 (12)	-5 (11)	-6 (12)
0.9	-6 (2)	-7 (2)	-7 (2)	-7 (2)	-4 (7)	-5 (6)	-6 (7)	-6 (6)	-4 (10)	-5 (10)	-5 (10)	-6 (10)
						$n = 1024, \delta = 4$						
0.6					-4 (7)	-5 (7)	-5 (7)	-6 (8)	-6 (19)	-5 (17)	-7 (18)	-7 (19)
0.7					-3 (9)	-4 (9)	-4 (9)	-5 (9)	-4 (16)	-4 (17)	-5 (16)	-6 (17)
0.8					-6 (10)	-6 (10)	-7 (10)	-6 (10)	-5 (18)	-6 (17)	-6 (17)	-7 (18)
0.9					-5 (9)	-5 (9)	-6 (9)	-6 (9)	-5 (14)	-5 (14)	-5 (14)	-5 (14)
						$n = 1024, \delta = 8$						
0.6					-5 (10)	-6 (10)	-6 (10)	-6 (10)	-10 (31)	-10 (28)	-11 (29)	-11 (30)
0.7					-4 (12)	-4 (12)	-5 (12)	-5 (12)	-7 (27)	-7 (28)	-8 (28)	-8 (28)
0.8					-6 (13)	-6 (13)	-7 (12)	-7 (13)	-7 (30)	-6 (29)	-7 (28)	-9 (31)
0.9					-7 (13)	-7 (13)	-7 (13)	-7 (13)	-7 (25)	-7 (25)	-7 (25)	-7 (26)

King, M. R. (2005) Fractal analysis of eight glacial cycles from an antarctic ice core, *Chaos Solitons & Fractals*, **25**, 5–10.

Knight, M. I. and Nunes, M. A. (2012) *nlt: a nondecimated lifting scheme algorithm*, R package version 2.1-3.

McCoy, E. J. and Walden, A. T. (1996) Wavelet analysis and synthesis of stationary long-memory processes, *Journal of Computational and Graphical Statistics*, **5**, 26–56.

Peng, C.-K., Buldyrev, S. V., Havlin, S., Simons, M., Stanley, H. E., and Goldberger, A. L. (1994) Mosaic organization of DNA nucleotides, *Physical Review E*, **49**, 1685.

Wuertz, D. et al. (2013) *fARMA: ARMA Time Series Modelling*, r package version 3010.79.

Table 18: Empirical estimator bias $(\times 100)$ after aggregating fractionally integrated series for a range of Hurst parameters featuring different degrees of missing observations to sampling intervals of size δ for three estimation methods. Numbers in brackets are the estimation errors' standard deviation.

	LoMPE				Peng				Wavelet			
							$n = 256, \delta = 2$					
H	5%	10%	15%	20%	5%	10%	15%	20%	5%	10%	15%	20%
0.6	-3 (4)	-2 (5)	-2 (5)	-3 (5)	-5 (10)	-5 (11)	-6 (10)	-7 (9)	-9 (26)	-10 (26)	-11 (28)	-7 (24)
0.7	-5 (5)	-5 (5)	-5 (5)	-5 (5)	-6 (10)	-6 (10)	-7 (11)	-8 (11)	-10 (29)	-10 (29)	-10 (29)	-13 (30)
0.8	-7 (6)	-7 (6)	-7 (6)	-8 (5)	-6 (12)	-7 (11)	-8 (12)	-9 (12)	-6 (26)	-6 (26)	-7 (28)	-9 (29)
0.9	-7 (6)	-8 (6)	-8 (6)	-8 (6)	-6 (12)	-8 (12)	-8 (12)	-9 (12)	-4 (28)	-6 (27)	-6 (27)	-9 (29)
							$n = 512, \delta = 2$					
0.6	-3 (3)	-3 (3)	-3 (3)	-3 (3)	-3 (8)	-3 (8)	-4 (8)	-4 (8)	-6 (18)	-5 (17)	-5 (16)	-6 (15)
0.7	-6 (3)	-6 (3)	-7 (3)	-7 (3)	-4 (7)	-5 (7)	-6 (7)	-6 (7)	-3 (15)	-4 (15)	-6 (15)	-6 (16)
0.8	-8 (4)	-8 (4)	-8 (4)	-9 (4)	-6 (9)	-6 (9)	-7 (10)	-8 (9)	-7 (19)	-7 (18)	-8 (18)	-9 (17)
0.9	-9 (4)	-9 (4)	-9 (4)	-10 (5)	-6 (9)	-7 (9)	-8 (9)	-9 (9)	-4 (14)	-6 (15)	-6 (14)	-7 (14)
							$n = 512, \delta = 4$					
0.6					-3 (12)	-3 (12)	-4 (11)	-4 (11)	-7 (31)	-4 (28)	-4 (28)	-7 (27)
0.7					-4 (10)	-5 (10)	-5 (9)	-6 (10)	-1 (27)	-3 (27)	-4 (27)	-4 (25)
0.8					-6 (13)	-6 (13)	-7 (14)	-7 (13)	-7 (31)	-7 (30)	-8 (30)	-9 (32)
0.9					-7 (11)	-7 (11)	-7 (11)	-8 (11)	-3 (24)	-4 (24)	-4 (25)	-5 (25)
							$n = 1024, \delta = 2$					
0.6	-4 (2)	-4 (2)	-4 (2)	-4 (2)	-3 (5)	-4 (5)	-4 (5)	-4 (5)	-4 (11)	-5 (11)	-6 (11)	-5 (11)
0.7	-6 (2)	-6 (2)	-7 (3)	-7 (3)	-4 (5)	-4 (5)	-5 (6)	-6 (6)	-3 (10)	-4 (10)	-4 (9)	-6 (11)
0.8	-8 (3)	-8 (3)	-9 (3)	-9 (3)	-4 (7)	-4 (7)	-5 (7)	-6 (7)	-3 (11)	-3 (10)	-5 (11)	-5 (11)
0.9	-9 (3)	-10 (3)	-10 (4)	-10 (4)	-4 (7)	-5 (7)	-6 (7)	-7 (7)	-5 (12)	-5 (11)	-6 (12)	-7 (11)
							$n = 1024, \delta = 4$					
0.6					-4 (7)	-3 (7)	-4 (6)	-4 (6)	-5 (17)	-6 (16)	-7 (16)	6 (17)
0.7					-4 (7)	-5 (7)	-6 (8)	-6 (8)	-3 (14)	-4 (15)	-5 (14)	-6 (16)
0.8					-4 (9)	-4 (9)	-5 (9)	-6 (9)	-3 (16)	-4 (16)	-4 (16)	-4 (15)
0.9					-4 (9)	-4 (10)	-5 (9)	-5 (9)	-5 (18)	-5 (17)	-6 (17)	-7 (18)
							$n = 1024, \delta = 8$					
0.6					-4 (9)	-4 (10)	-4 (9)	-4 (9)	-8 (28)	-10 (27)	-9 (27)	-10 (31)
0.7					-4 (10)	-5 (10)	-6 (10)	-5 (10)	-4 (23)	-4 (24)	-5 (23)	-6 (25)
0.8					-4 (13)	-4 (12)	-5 (13)	-5 (13)	-2 (26)	-2 (26)	-3 (26)	-2 (25)
0.9					-4 (13)	-4 (13)	-5 (13)	-5 (13)	-6 (32)	-6 (31)	-7 (32)	-7 (32)

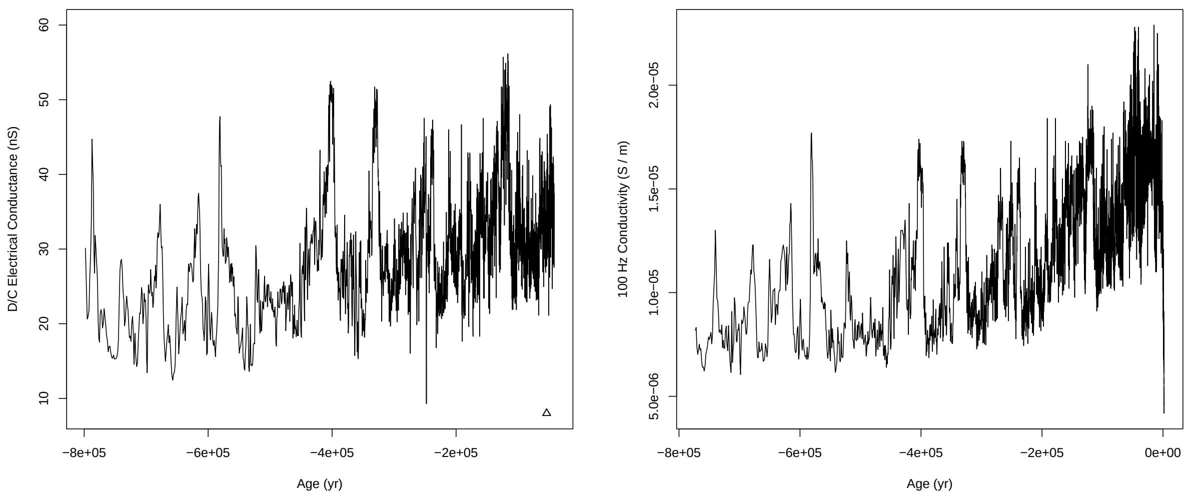

Figure C.1: The `D/C` ice core series (left) and the `dielectric` ice core series (right).

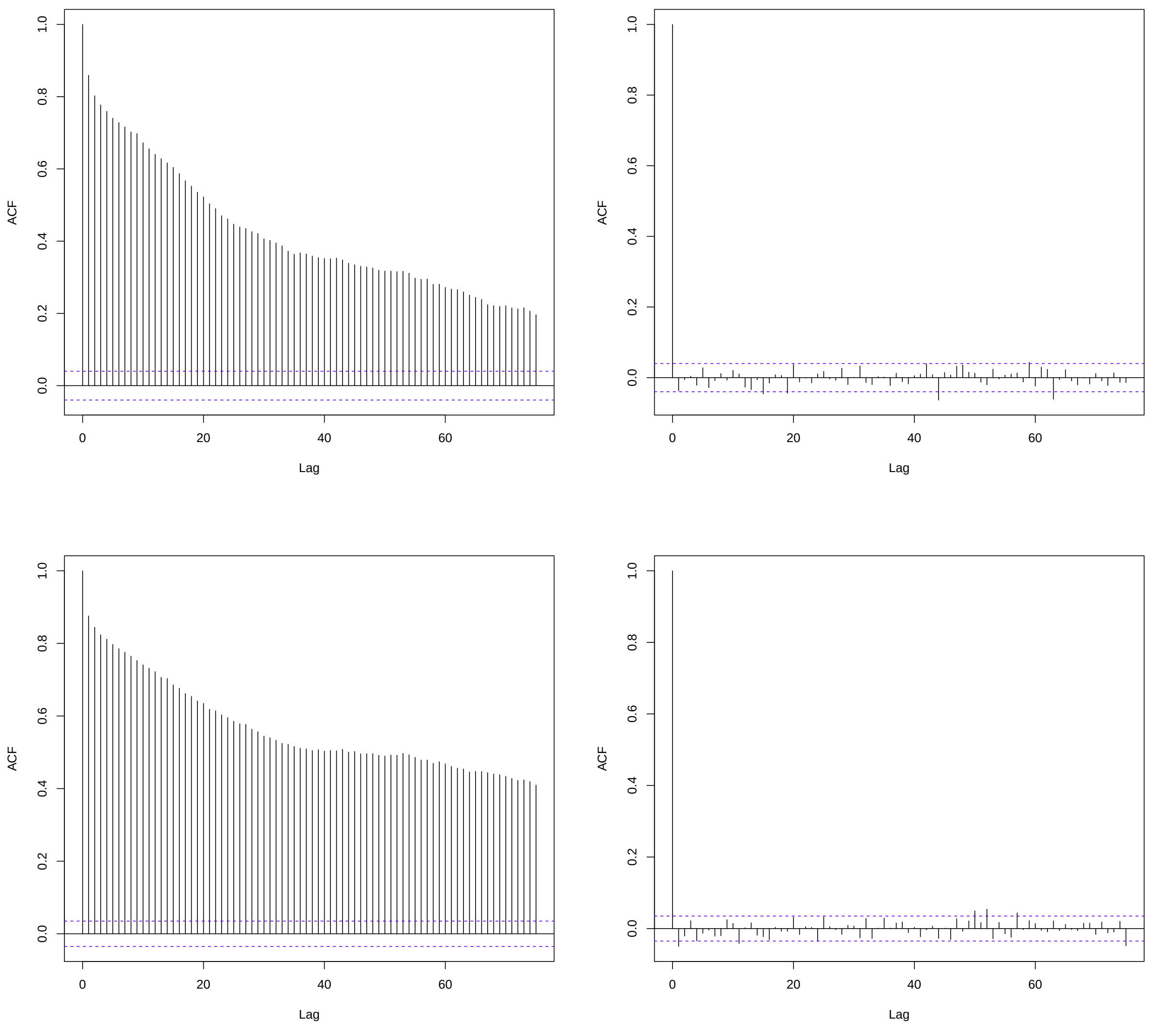

Figure C.2: Autocorrelation of the electric conductance ice core data from Figure C.1 before and after LOCAAT transformation. Top: the `D/C` ice core series; bottom: the `dielectric` ice core series.
